# Host preferences support the prominent role of *Hyalomma* ticks in the ecology of Crimean-Congo hemorrhagic fever

**DOI:** 10.1371/journal.pntd.0006248

**Published:** 2018-02-08

**Authors:** Jessica R. Spengler, Agustin Estrada-Peña

**Affiliations:** 1 Viral Special Pathogens Branch, Centers for Disease Control and Prevention, Atlanta, GA, United States of America; 2 Department of Animal Health, Faculty of Veterinary Medicine, University of Zaragoza, Zaragoza, Spain; Vienna, AUSTRIA

## Abstract

Crimean-Congo hemorrhagic fever virus (CCHFV) is a tick-borne zoonotic agent that is maintained in nature in an enzootic vertebrate-tick-vertebrate cycle. *Hyalomma* genus ticks have been implicated as the main CCHFV vector and are key in maintaining silent endemic foci. However, what contributes to their central role in CCHFV ecology is unclear. To assess the significance of host preferences of ticks in CCHFV ecology, we performed comparative analyses of hosts exploited by 133 species of ticks; these species represent 5 genera with reported geographical distribution over the range of CCHFV. We found that the composition of vertebrate hosts on which *Hyalomma* spp. feed is different than for other tick genera. Immatures of the genus *Hyalomma* feed preferentially on species of the orders Rodentia, Lagomorpha, and the class Aves, while adults concentrate mainly on the family Bovidae. With the exception of Aves, these hosts include the majority of the vertebrates consistently reported to be viremic upon CCHFV infection. While other tick genera also feed on these hosts, *Hyalomma* spp. almost completely concentrate their populations on them. *Hyalomma* spp. feed on less phylogenetically diverse hosts than any other tick genus, implying that this network of hosts has a low resilience. Indeed, removing the most prominent hosts quickly collapsed the network of parasitic interactions. These results support the intermittent activity of CCHFV foci: likely, populations of infected *Hyalomma* spp. ticks exceed the threshold of contact with humans only when these critical hosts reach adequate population density, accounting for the sporadic occurence of clinical tick-transmitted cases. Our data describe the association of vertebrate host preferences with the role of *Hyalomma* spp. ticks in maintaining endemic CCHFV foci, and highlight the importance of host-tick dynamics in pathogen ecology.

## Author summary

Crimean-Congo hemorrhagic fever virus (CCHFV), a cause of severe hemorragic disease in humans, is maintained in nature in a tick-vertebrate-tick enzootic cycle characterized by silent persistence of endemic foci with intermittent epidemics. Most studies support ticks of the genus *Hyalomma* as the main CCHFV vectors, but some laboratory reports and field studies also allude to other tick species that may be responsible for virus circulation. Here we converted the tick-host interactions of 133 species of ticks with reported geographical distribution over the range of CCHFV into a network of relationships. By a series of network analyses, we found that immatures of the genus *Hyalomma* are unique in their hosts preferences among the examined tick genera. Immatures of the genus *Hyalomma* concentrate on rodent and rabbit hosts, which most efficiently support CCHFV maintenance and transmission by ticks in nature. Based on these data, we formulate the hypothesis that the ecological relationships between *Hyalomma* spp. and their hosts form a delicate equilibrum that differentiates human epidemics from periods of silent CCHFV maintenance.

## Introduction

Crimean-Congo hemorrhagic fever (CCHF) is a tick-borne zoonotic disease seen exclusively in humans that can progress from mild, non-specific signs to a severe and fatal hemorrhagic disease. The etiologic agent, Crimean-Congo hemorrhagic fever virus (CCHFV; family *Nairoviridae*, genus *Orthonairovirus*), is transmitted to humans predominantly via tick bites, but may also be transmitted nosocomially or by handling tissues from viremic animals (e.g., in abattoirs). As non-human vertebrate hosts do not develop clinical signs [1], maintenance in nature is largely silent. Recent reviews summarize the current knowledge about serology in animals [2], routes of transmission [3], and the tick species unambiguously involved in CCHFV circulation in natural and permanent foci [4].

Ticks are vectors and reservoirs for CCHFV; vertebrates act as a bridge, transmitting the virus to new generations of ticks. Infected vertebrates develop a short viremia [1], and virus is transmitted to ticks feeding on viremic hosts, or through co-feeding with infected ticks (demonstrated by [5,6]), which release the virus into the feeding cavity where other uninfected ticks feed. Most reports support ticks of the genus *Hyalomma* as the main CCHFV vector, but laboratory and field studies allude to other tick species that may also be responsible for virus circulation [4]. To date, interactions between CCHFV and the tick are not well known, including hypothetical molecular factors that could regulate infection and viral dissemination through a variety of physiological and anatomical barriers in the tick [7]. While molecular interactions are an obvious research target to explain the prominence of *Hyalomma* spp. in CCHFV maintenance and transmission, other non-molecular relationships may also be involved or even predominate. Some ticks of other genera may act as efficient CCHFV vectors under adequate laboratory conditions (e.g., as reviewed in [4]), raising the question about the importance of intimate molecular relationships between tick and virus versus the simple ecological interactions of ticks and key hosts in supporting silent CCHFV foci. In other words, the dynamics of CCHFV transmission may be driven by purely ecological factors and not depend on molecular compatibility.

Most tick species are not restricted in range by their hosts; rather, climate is the main driver of their distribution patterns [8]. With the exception of some monoxenic species, ticks regularly feed on a wide range of hosts. Permanent foci of some tick-transmitted pathogens are restricted to the range of key reservoirs or vectors. For example, *Borrelia burgdorferi* s.l. is intimately linked with the tick genus *Ixodes*; *Babesia bovis* is transmitted exclusively by boophilid ticks; and tick-borne encephalitis virus is restricted to rodents reservoirs [9–11]. Likewise, CCHFV could conceivably circulate only in areas that support a delicate equilibrium of abundance and composition of appropriate hosts for the ticks.

We hypothesized that a key factor for maintaining CCHFV foci is a precise combination of host species that feed the ticks, thereby amplifying infection in the tick population. A network-based analysis, comprising relationships among ticks and hosts connected by pairwise relations, enables the reconstruction of associations between ticks and key vertebrates in the circulation of a pathogen. This kind of ecological modeling is made possible by an extensive toolbox developed for network research (see, for example, [12–14]). The structural properties of tick-vertebrate networks reveal new insights into the associations linking ticks and hosts that are key for supporting permanent CCHFV foci.

Here we aimed to compare the combinations of hosts parasitized by ticks colonizing the reported range of CCHFV, focusing on explicit relationships between ticks and the hosts reported to support viremia. We explicitly tested the phylogenetic diversity and the centrality (i.e., the relative importance in the network of connections) of the groups of hosts used by these ticks, the resilience of the networks to the removal of hosts, and the existence of clusters of tick-host interactions. We used these findings to elaborate on the specific relationships of *Hyalomma* spp. with their hosts and determine if these relationships differ from those of other ticks. We also attempted to identify the key factors shaping the circulation of CCHFV mainly by *Hyalomma* spp. ticks, and ascertain how these specific combinations drive unstable foci of the virus.

## Materials and methods

### Acquiring data on tick hosts

A species-by-species analysis of the tick-host relationships is not possible, because i) some ticks species are underreported (e.g., prevalence, host preference) in the literature and therefore an evident bias in the number of hosts is expected; and ii) the immatures of some ticks are difficult to identify, leading to the reporting of host species that support improperly identified ticks. Following the same reasoning, the analysis of the relationship between ticks and specific host species is not possible; some vertebrates may be very poorly surveyed (because they are rare, difficult to trap, or protected, etc.), which would undoubtedly bias the holistic approach. We therefore used the data on families of hosts for each species of tick, as reported by [15]. The geographical range refers to the complete Palearctic and Afrotropical regions, which are the territories in which CCHFV circulation has been described. Then, data were summarized at the level of tick genera.

### Network development, calculation of centrality, and cluster analysis

An estimation of the relative importance of each family of vertebrates was developed in the context of a network of tick-host relationships, at the level of tick genera and life stages (larvae, nymphs, or adults), similar to network approaches commonly performed in other scientific fields [16–18]. A network is a construct that reflects organisms (nodes) that interact in any way (links). In our approach, nodes are ticks and vertebrates, and links display the reported finding of a given tick and life stage on a vertebrate of a given family. It is thus a directed network, since ticks have been recorded *on* hosts. The basic index of a network is the weighted degree (WD), defined as the weighted number of times a group of hosts is recorded for the complete set of tick genera [19]. We calculated the betweenness centrality (BNC, [20]), an index that explains how important a node is in linking several other nodes of either ticks or vertebrates. The ecological significance of the index in our application is immediate: BNC is higher for families of vertebrates that are predominantly used as hosts by several genera of ticks. Separate calculations of BNC for each genus and life stage of ticks give a comparitive overview of the relative importance of the hosts. Clusters of the network were calculated using the algorithm of Neumann [21]. A cluster is a group of nodes that interact more among themselves than with other nodes in the network. Clusters have importance in this context because they reflect groups of vertebrates among which ticks interact more commonly, thus displaying an ecological relationship. Network calculations were done for the complete dataset (to capture the structure of the complete network of interactions), as well as separately for every genus and stage of ticks (to understand the ecological relationships of every tick genus independently of the rest).

### Phylogenetic relationships among hosts

For each genus and stage of ticks, we calculated the genetic richness of the exploited hosts using Faith’s phylogenetic distance (PD). This metric is based on the sum of distances of the branches that link any pair of families in the phylogenetic tree [22], and is used to determine if the different genera and stages of ticks parasitize phylogenetically narrow or wide host ranges. PD is an adequate estimate in this context, and supersedes simple measures of host variability based solely on the number of different taxa that serve as tick hosts. We first obtained the phylogenetic tree of the families of hosts, as available in the Open Tree of Life (OTL, https://tree.opentreeoflife.org). The OTL is a repository of phylogenetic trees and produces synthetic trees for a broad range of organisms. It can be accessed through its API to obtain portions of the complete phylogenetic tree stored in the repository. We used a script in the R programming environment to query OTL for the phylogenetic pattern of the families of hosts used by the ticks examined in this study. The resulting tree (see S1 Fig) contained data on the phylogenetic relationships of 92 families of vertebrates and was suitable for obtaining estimates of the relative branching of the vertebrates utilized by each genus of tick, but had no calibrated date because it was a synthetic tree. The remaining 40 families of hosts had no information in OTL, and the available information in GenBank was too fragmented to be combined with the already built tree.

### Resilience of the networks of ticks and hosts

We evaluated the resilience of the network of tick-vertebrate relationships separately for each tick genus and life stage to understand how random or directed attacks could affect its stability. Resilience of a host-parasite network is an important feature emanating from the network approach, and can be evaluated by removing the hosts either randomly or based on their BNC order in the network. Resilience is measured in terms of the probability of network collapse; the removal of key hosts may lead the network to break down without further links of the parasites to the remaining hosts. We built and obtained indexes of the network structure on the R programming environment [23] using the igraph [24], bipartite [25], and picante [26] packages. The resilience of the networks of each genus and stage of ticks after recursive removal of host nodes was evaluated with the package NetSwan for R [27]. Visualization of the networks was done in Gephi v0.91 [28].

## Results

### Developing a tick-host interaction database for ticks implicated in CCHFV transmission

To analyze the relationships between ticks and hosts with evidence of a potential role in CCHFV circulation, we compiled a list of tick species distributed over the geographical range of CCHFV. This range includes the complete Afrotropical region, the Mediterranean Palaearctic region, and most of Central Asia from the Turkish steppes to India. We focused on 5 tick genera: *Amblyomma*, *Dermacentor*, *Hyalomma*, *Ixodes*, and *Rhipicephalus*, which contain species that have been implicated in CCHFV transmission through either studies of natural foci or in the laboratory. All species in the genera were included in the analysis, but not every species included has been reliably linked to CCHFV transmission. Our dataset contained 22 species of genus *Amblyomma*, 2 of *Dermacentor*, 18 of *Hyalomma*, 44 of *Ixodes*, and 47 of *Rhipicephalus*, with a total of 1591 pairs of reported associations between the 133 tick species and 132 families of hosts. Phylogenetic calculations were performed on 92 vertebrate families. The network construct had a total of 147 nodes (genera and stages of ticks, families of hosts) and 553 links. The complete list of ticks and hosts is included as S1 Table. Values of BNC for each family of hosts (converted to the range 0–100 to improve comparisons) are included in S1 Data.

### Clustering and centrality values of CCHFV-associated host-tick networks

**Fig 1:**
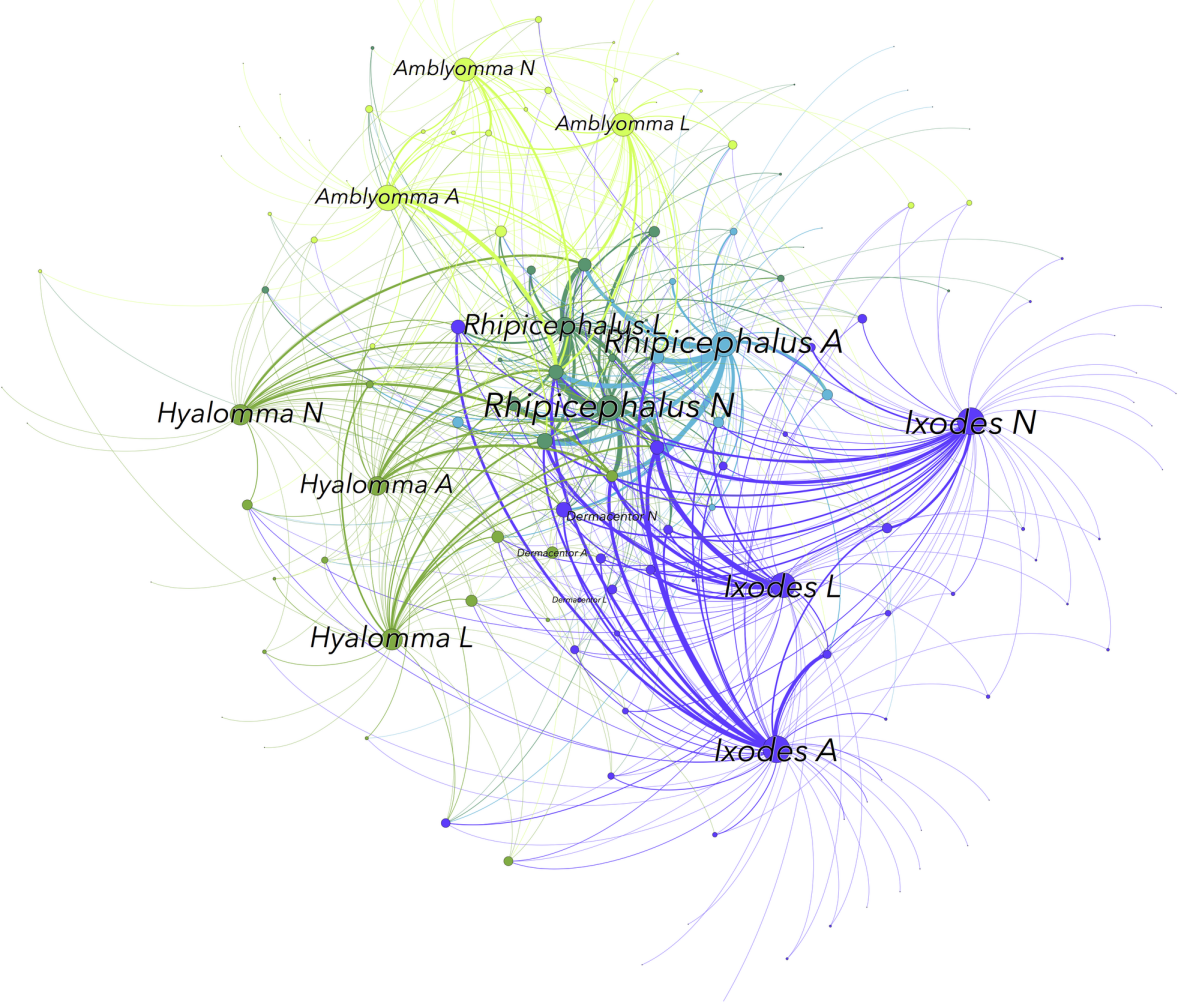
The network of interactions between tick genera and life stages (L, larvae; N, nymphs; A, adults) and the families of hosts. Only the genera and stages of ticks are labeled to improve readabilty. S2 Fig includes the same figure with all the nodes labeled. Nodes are sequentially colored to represent clusters, with nodes of the same color belonging to the same cluster. The size of each node is porportional to its betweenness centrality (BNC), and the size of the label is proportional to its weighted degree (WD). The width of the links is proportional to the weighted number of interactions recorded between hosts and ticks.

The network construct provides a representation of the tick-host relationships, enabling research on the principles behind complex interactions. Fig 1 displays the network and its clusters, explicitly describing the sets of nodes that interact more among themselves than with others (see also S2 Fig). Up to 5 groups or clusters of organisms can be detected, denoting dominant interactions between sets of ticks and vertebrates. Interestingly, each cluster was formed by the 3 life stages of the same genus of ticks, except for *Dermacentor* and the adults of genus *Rhipicephalus*. The adults of genus *Dermacentor* appeared in the same cluster as adults of genus *Hyalomma*, and the immatures clustered with immatures of the genus *Ixodes*. The adults of *Rhipicephalus* formed their own cluster of interacting organisms.

The overview of the relationships between ticks and vertebrates (Fig 1) shows 54 families of hosts (36.73% of total) that are only slightly important, in terms of BNC, for supporting the network of ticks; these families appear at the periphery of the network. Most *Amblyomma* species use hosts of the classes Reptilia and Aves, and, in a large proportion, the family Bovidae. Patterns of diversity for *Amblyomma* spp. are high, with 36, 35, and 37 vertebrate families used by larvae, nymphs, and adults, respectively. A similar pattern is seen for *Hyalomma* spp.: the larvae, nymphs, and adults use 41, 39, and 37 vertebrate families. Most of the vertebrates families used as hosts by *Hyalomma* spp. are small endotherms, including members of the orders Rodentia, Lagomorpha, and Artiodactyla, and the class Aves.

Leporidae and Muridae had BNC values of 1222 and 583 for larvae of the genus *Hyalomma*, and 1332 and 581 for nymphs, demonstrating the importance of these vertebrate families in supporting ticks of this genus. In contrast, Muridae had a BNC value of only 76 for larvae of the genus *Amblyomma*, and neither Muridae nor Leporidae was parasitized by *Amblyomma* nymphs. Adults of both *Hyalomma* and *Amblyomma* spp. feed on ungulates (family Bovidae, BNC = 460 for *Hyalomma* and 1544 for *Amblyomma*). Interestingly, adults of the genus *Hyalomma* also utilized Leporidae (BNC = 248).

The pattern was completely different for *Ixodes* spp., the ticks that feed on the widest variety of vertebrates, including 51 families exploited by larvae, 59 by nymphs, and 56 by adults. Every stage of this tick genus was widely and loosely distributed over a wider variety of hosts than other tick genera.

*Rhipicephalus* spp. were highly eclectic in host preference, and the 3 life stages of this genus do not cluster in a discrete group, preferring Bovidae, Canidae, and a variety of Carnivora hosts during the larval, nymphal, and adult stages, respectively. Results for the genus *Dermacentor*, with only 2 species included in analysis, showed that immatures mainly parasitize Muridae, while adults concentrated on Suidae.

### Phylogenetically narrow host associations of *Hyalomma* spp. ticks

To evaluate whether ticks of each genus were restricted to a wide or narrow host range according to PD of vertebrate families, we aimed to capture the PD of the hosts supporting every genus and stage of the ticks examined. For example, a tick genus may use several vertebrate families that are phylogenetically very related and thus uses a narrow range of hosts, or utilize a few vertebrate host families that are phylogenetically distant, thus covering a broad range of phylogenetic diversity. Analysis resulted in a tree containing data on the phylogenetic relationships of 92 vertebrate families (S1 Fig). We found that the PD of the different genera and stages of ticks varied highly (summarized in Table 1). With the exception of the genus *Dermacentor*, the genus *Hyalomma* showed the lowest PD, even though *Hyalomma* spp. can parasitizes a number of host families similar to other tick genera. Although the low PD for genus *Dermacentor* was notable, only 2 species of this genus were included in the study, compared to 18 species of *Hyalomma* ticks.

**Table 1:** Genera and life stages of ticks studied, with the phylogenetic diversity of their hosts (PD) and the number of vertebrate host families recorded. FR, family richness with phylogenetic information.

Genus and stage	PD	FR
***Amblyomma*** (Adults)	4.703	28
***Amblyomma*** (Larvae)	4.207	28
***Amblyomma*** (Nymphs)	4.432	23
***Dermacentor*** (Adults)	2.596	7
***Dermacentor*** (Larvae)	1.826	6
***Dermacentor*** (Nymphs)	1.714	9
***Hyalomma*** (Adults)	2.896	26
***Hyalomma*** (Larvae)	3.816	31
***Hyalomma*** (Nymphs)	3.777	28
***Ixodes*** (Adults)	4.888	39
***Ixodes*** (Larvae)	4.699	36
***Ixodes*** (Nymphs)	5.526	41
***Rhipicephalus*** (Adults)	4.576	42
***Rhipicephalus*** (Larvae)	5.138	35
***Rhipicephalus*** (Nymphs)	5.364	43

**Fig 2:**
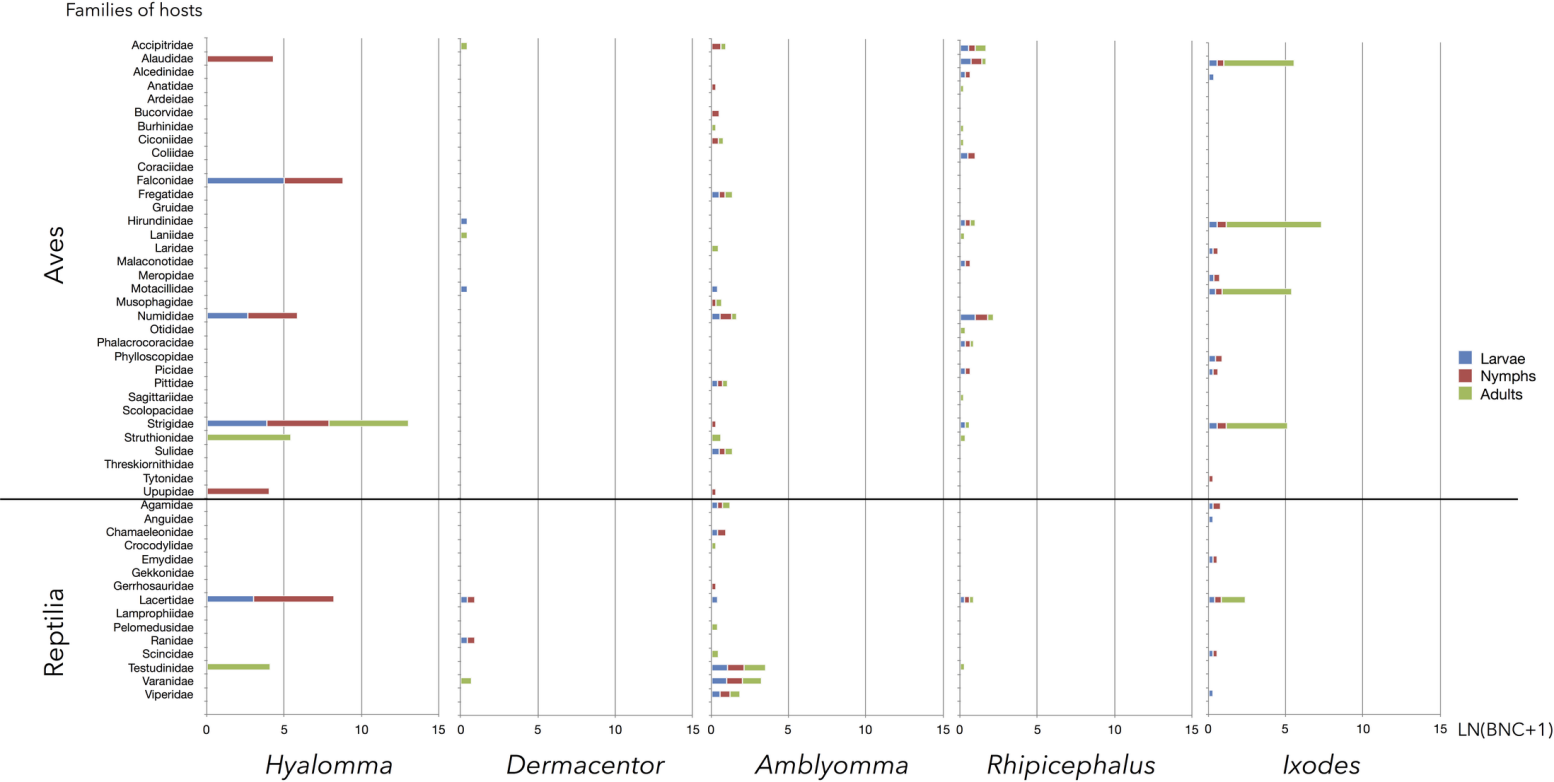
The betweenness centrality (BNC, converted to natural log of value + 1) of Aves and Reptilia hosts (alphabetically, at left) for each group of tick genera and life stages (bottom). A, adults; L, larvae; N, nymphs.

**Fig 3:**
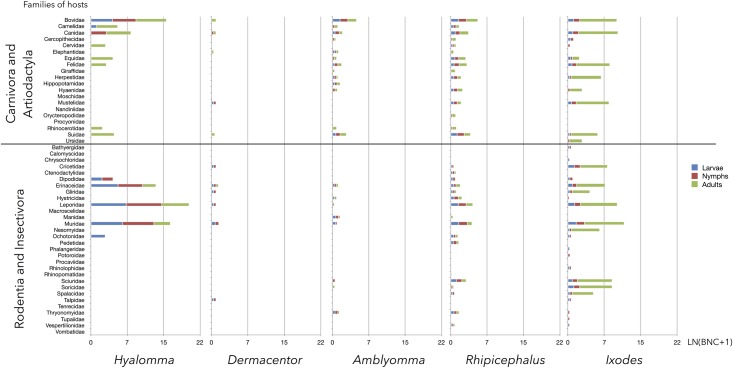
The BNC (converted to natural log of value + 1) of the families of Rodentia, Insectivora, Carnivora, and Artiodactyla hosts (alphabetically, at left) for each group of tick genera and life stages (bottom). A, adults; L, larvae; N, nymphs.

From the results in Table 1 and Fig 1, we summarized both BNC and PD for each host family, tick genus, and tick life stage (Figs 2 and 3). *Hyalomma* was i) the genus with lowest phylogenetic diversity of hosts during all 3 developmental stages; and ii) the only genus the immatures of which concentrate on Leporidae and Muridae hosts while the adults fed mainly on Bovidae. This was demonstrated by high BNC values of these vertebrate families for *Hyalomma* spp., which was not seen for other tick genera. Most important in this context, adult *Hyalomma* spp. ticks have also been found associated with Leporidae hosts. Additionally, a few Aves species have a relatively high importance as hosts for *Hyalomma* spp., and are involved mainly in circulating immature ticks.

### Low network resilience of *Hyalomma* spp. ticks based on removal of key hosts

Many complex systems display a surprising degree of error tolerance. However, networks with prominent hubs have low resilience and are extremely vulnerable to attacks (that is, to the selection and removal of a few nodes that play a vital role in maintaining the connectivity of the network) [29]. We aimed to capture the behavior of the networks of each tick genus and stage after recursive removal of host nodes. Resilience is an important feature of the ecological networks in which some organisms (ticks) depend on the presence of others (hosts) that may be key for the circulation of the parasite. After removing each node, the complete network was recalculated and its connectivity was re-evaluated. The percent of connectivity loss was the key measure of the resilience of the network to attack (random attack, or removing in order of decreasing BNC or decreasing WD). These calculations could not be done for the genus *Dermacentor*, because the connectivity dropped unrealistically after the removal of only a few nodes due to the limited number of species studied.

**Fig 4:**
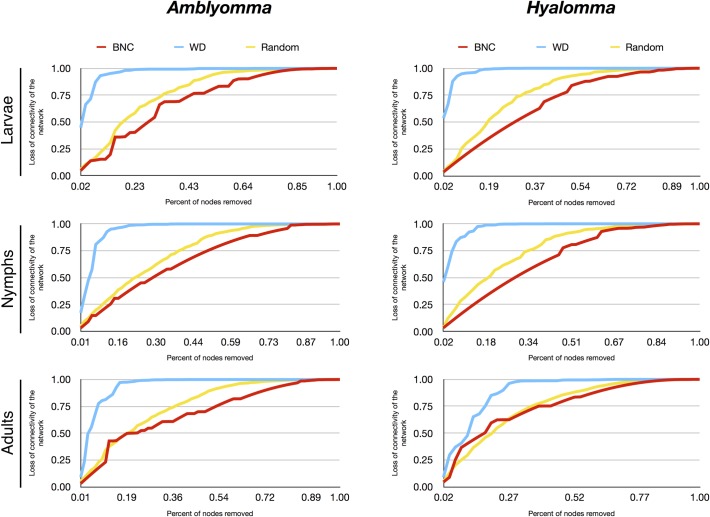
The loss of connectivity in the networks of larvae, nymphs, and adults of the tick genus *Amblyomma* and the genus *Hyalomma*. Loss of connectivity is calculated after removing hosts randomly (Random, yellow), or after removing hosts in decreasing order of their BNC (red) or of their WD (blue).

**Fig 5:**
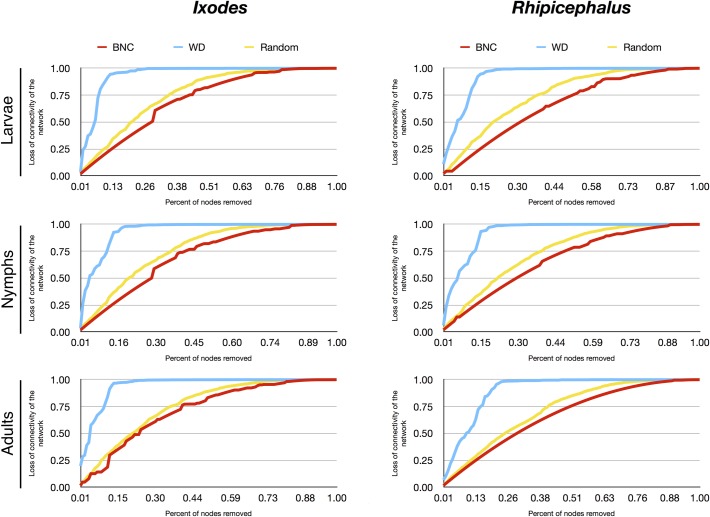
The loss of connectivity in the networks of larvae, nymphs, and adults of the genus *Ixodes* and the genus *Rhipicephalus*. Loss of connectivity is calculated after removing hosts randomly (Random, yellow), or after removing hosts in decreasing order of their BNC (red) or of their WD (blue).

The networks of every stage and genus of tick analyzed were very resilient to random removal of hosts, all of them resulting in a loss of ~75% of connectivity after the removal of 50% of the nodes according to decreasing BNC of hosts (Figs 4 and 5). Random removal of host nodes promoted higher loss of connectivity in every network. However, lowest resilience of the networks was obtained when they were subjected to removal of hosts according to their WD; *Hyalomma* larvae and nymph results were deeply affected by removing as few as 2% of hosts with the highest WD. Removing Bovidae, Muridae, and Leporidae, host families that have been reported to develop consistent viremia upon CCHFV infection, resulted in almost complete collapse of the *Hyalomma* spp. larvae and nymph networks. Removing Bovidae, Leporidae, and Suidae promoted a ~50% loss of connectivity for *Hyalomma* spp. adults. Although immatures of the genus *Amblyomma* were also affected by the removal of hosts according to their WD, these ticks were most affected by removing lizards and amphibians, which are not known to be involved in the CCHFV lifecycle. The ecological significance of these findings is that *Amblyomma* spp. larvae and *Hyalomma* spp. immatures depend on key vertebrate families as hosts. Notably, the hosts on which *Hyalomma* spp. mostly depend are of pivotal importance for CCHFV circulation.

## Discussion

The main vectors of CCHFV are considered to be ticks of the genus *Hyalomma*. However, viral transmission has been confirmed under laboratory conditions in ticks of other genera co-occurring with *Hyalomma*. What differentiates CCHFV vector capacity of *Hyalomma* spp. from that of other ticks, including those capable of virus transmission, is not clear. We examined ecological factors to investigate whether special characteristics of the communities of hosts used by each tick genus could have a role in CCHFV epidemiology and distribution. The main aim was to describe the ecological relationships among ticks and vertebrates, and to discern if distinct interactions could capture the prominent role of the tick genus *Hyalomma* in CCHFV circulation.

CCHFV is well known to circulate through the 3 stages of the tick developmental cycle. The virus persists in ticks through the developmental stages by transstadial survival, and is maintained in new tick generations by transovarial passage [5,30]. Brief viremia in vertebrate tick hosts is the bridge by which the virus accesses other ticks. CCHFV can also infect ticks by transmission among co-feeding ticks, a process by which uninfected ticks feeding in close proximity with infected ticks on non-viremic hosts become infected [31]. Field surveys systematically report clumped distributions of ticks on vertebrates [32]: a few hosts carry large numbers of ticks aggregated in close proximity, while most of the hosts carry few or no ticks. This is of particular interest for the co-feeding mechanism, since the ticks concentrate highly on small mammals, which develop longer viremia and serve as hosts for immature ticks (reviewed by [1]). Since these groups of hosts are important for CCHFV transmission, the preferences for them would concentrate most of the tick populations on key carriers for viral circulation, and increase the probability of CCHFV transmission by co-feeding.

Here, based on network analyses of CCHFV associated tick-host relationships, we found that every genus of ticks examined, except *Dermacentor*, had its own set of preferred hosts; the exception was probably because only 2 *Dermacentor* species were included in the dataset. Also, our results suggest that *Rhipicephalus* adults prefer a set of hosts completely different from those exploited by immatures of the same genus. Most importantly, we identified unequivocal host relationships of *Hyalomma* genus ticks. Immatures of this tick feed on only a few members of Rodentia, Lagomorpha, and Aves, while adults are tightly associated with large ungulates, with lower but still prominent preferences for Lagomorpha and Suidae.

Rodents, lagomorphs, and ungulates, but not birds (with the exception of ostriches [33]), have been demonstrated to develop viremia for a variable, but brief, period of time [1,34,35]. Values of centrality of these hosts show that they are of major importance for *Hyalomma* spp. Other genera of ticks may use similar groups of hosts, but are also widely distributed over many other host families. In other words, the immatures of the genus *Hyalomma* tend to concentrate and over-aggregate on vertebrates that have been shown to be important in CCHFV transmission, even if viremia in these hosts is transient. Other genera of ticks feed on these same hosts, but also on a wide array of alternative hosts that are not known to circulate the virus. This feature has been called the dilution effect for other tick-transmitted pathogens, like *B*. *burgdorferi* [36,37]. While the effect seems not to be universal [38,39], an adequate balance of carrier and non-carrier hosts that ticks can use would attenuate the prevalence of a pathogen in ticks. We explicitly propose that the particular associations of *Hyalomma* spp. with their hosts are responsible for the prominent role of this tick genus in CCHFV circulation. It must to be noted that, depending on abundance of various host species in a given region, ticks of other genera could theoretically circulate the virus also, as demonstrated in laboratory protocols [4].

This scenario of immature *Hyalomma* ticks over-aggregating on some key vertebrates while adults infest large ungulates (which may be also viremic) has been reported as the main driver of CCHF epidemics [40]. Abandoned agricultural areas become populated by large patches of natural flora, facilitating shelter for rodents, birds, wild suids, and wild ungulates. We hypothesize that the overpopulation of these key hosts increases the abundance of *Hyalomma* spp. ticks, which could fuel CCHFV prevalence rates in the vectors in a feedback mechanism. As more ticks become infected, the probability of infecting reservoirs and naïve ticks and of transmittng the virus to humans increases.

Further analyses suggest that *Hyalomma* spp. ticks are associated with a phylogenetically narrow spectrum of hosts, accounting for the low resilience of the network of hosts for this tick genus. The lowest PD value was obtained for genus *Hyalomma* together with *Dermacentor*, but the result for the latter was considered biased, since only 2 *Dermacentor* species were included in this study. Other genera of ticks, including *Ixodes*, *Amblyomma*, and *Rhipicephalus*, had significantly higher values of host PD, suggesting that these ticks feed on a much wider range of hosts. In other words, the lack of significant host preference for the immatures of these tick genera would result in greater stochasticity in tick abundance, affecting the circulation of the virus.

CCHFV epidemiology is characterized by silent persistence of virus foci with intermittent epidemics. When abundance of key hosts is low, *Hyalomma* tick populations could also remain low, enough to further circulate CCHFV (probably only by transovarial passage [31]), but well below the threshold (R_0_) necessary to break the barrier of contact with humans. Small changes in the composition of vertebrate hosts could slighlty increase the value of R_0_, leading to the few CCHF cases reported annually in endemic countries [41]. Expansion of key host populations would lead to CCHF epidemics. It is necessary to stress that the only data about CCHFV distribution come from the detection of human clinical cases. Therefore, no information exists about tick densities or serology in hosts for areas where the virus circulates at levels below the epidemic threshold. When the key hosts for CCHFV circulation are absent, immature *Hyalomma* spp. ticks would use other hosts that are not viremic, deeply affecting the prevalence of the virus in these vectors.

The approach of this study is purely ecological and is based on the tenets of the network theory, which has deep roots in social behavior [42], links among computers [43], or mutualism between plants and pollinators [44,45]. An unbalanced representation of the tick-host interactions could constrain the results of this development, since poorly collected species could introduce a bias in the total number of records. We, however, evaluated the strength of associations between partners using a purposely inclusive systematic division of ticks (genera) and hosts (families) to prevent the noise generated by undersampled species, together with robust markers representing the relationships in directed networks [46]. This approach guarantees a minimum bias in indexes of the network but not in PD estimations. Furthermore, this approach reduces potential for biogeographical bias based on co-distribution of both tick and hosts in cluster analysis, and supports that the reported cluster formation is derived from an acual preference towards particular vertebrates.

A species-by-species analysis of tick-host relationships is not possible here, as detailed earlier. Our broad approach does not account for CCHFV strains (e.g., AP92, lineage Europe 2) that, in addition to circulation by *Hyalomma* spp. ticks [47], have been suggested to be circulated predominantly by other species such as those of the genus *Rhipicephalus* [48]. It should be noted, however, that no proof of the vectoral capacity of *Rhipicephalus* spp. ticks for strain AP92 has ever been obtained under adequate laboratory conditions [2]. A broad approach also prevents the ability to delineate diverse viral lineages circulated by different species of *Hyalomma* in our analyses. However, these relationships, which are likely due to overlapping geographical ranges of both ticks and viral strains, do not affect the observations detailed here. Analyses are based on inclusion of all tick reports, irrespective of *Hyalomma* species, and serves as a broad investigation on what differentiates *Hyalomma* ticks ecologically from other genera of potential CCHFV tick vectors.

While our approach is validated by field data supporting the importance of a critical combination of hosts that coexist during the life cycle of *Hyalomma* ticks, we must take into account the complete lack of data regarding the intimate molecular relationships of CCHFV with the tick. The tick gut represents the first barrier against pathogens, and the gut cell membrane is the key to dissemination of the virus into the body of the vector. The need to conduct these studies under high biocontainment has precluded the understanding of basic mechanisms that CCHFV uses to enter the tick gut and to disseminate to salivary glands for further circulation. While the ecological hypothesis that we outlined here provides a meanignful interpretation of CCHFV dynamics in the field, the relationships between the molecular machinery of the virus and the tick as an environment must be understood.

Our current knowledge on CCHFV distribution has been gathered from reported human clinical cases, which provide a fragmented picture of the much wider geographical range of the virus. Surveying the virus in wild hosts and questing ticks, together with an extensive record of tick-host relationships, is urgently needed to update exisiting data about this potentially lethal agent. Viral foci must also be associated with adequate definitions of the environmnetal niche to make sense of the elusive behaviour of the so-called silent foci. These studies, together with a deeper knowledge of the molecular mechanisms shaping the virus-vector interactions, are esential to identify the routes of CCHFV circulation and the exposed populations, and to outline adequate preventive mesaures.

## Supporting information

S1 Fig The phylogenetic tree of 92 vertebrate families that have been reported as hosts for the ticks included in the analysis, obtained from the Open Tree of Life (https://tree.opentreeoflife.org, accessed August, 2017). (TIF)

S2 Fig The network of interactions between different tick genera and life stages (L, larvae; N, nymphs; A, adults) and host families. Nodes are sequentially colored to represent clusters, with nodes of the same color belonging to the same cluster. The size of each node is porportional to its betweenness centrality (BNC), and the size of the label is proportional to its weighthed degree (WD). The width of the links is propotional to the number of interactions recorded between hosts and ticks. The image can be resized and magnified on the screen for optimum readability of the names of host families with low WD.
(TIF)

S3 Fig Plot of betweenness centrality (BNC) values for *Amblyomma* larvae. The size of the circles is proportional to BNC values, recoded on the interval 0–100.
(TIF)

S4 Fig Plot of betweenness centrality (BNC) values for *Amblyomma* nymphs. The size of the circles is proportional to BNC values, recoded on the interval 0–100.
(TIF)

S5 Fig Plot of betweenness centrality (BNC) values for *Amblyomma* adults. The size of the circles is proportional to BNC values, recoded on the interval 0–100.
(TIF)

S6 Fig Plot of betweenness centrality (BNC) values for *Dermacentor* larvae. The size of the circles is proportional to BNC values, recoded on the interval 0–100.
(TIF)

S7 Fig Plot of betweenness centrality (BNC) values for *Dermacentor* nymphs. The size of the circles is proportional to BNC values, recoded on the interval 0–100.
(TIF)

S8 Fig Plot of betweenness centrality (BNC) values for *Dermacentor* adults. The size of the circles is proportional to BNC values, recoded on the interval 0–100.
(TIF)

S9 Fig Plot of betweenness centrality (BNC) values for *Hyalomma* larvae. The size of the circles is proportional to BNC values, recoded on the interval 0–100.
(TIF)

S10 Fig Plot of betweenness centrality (BNC) values for *Hyalomma* nymphs. The size of the circles is proportional to BNC values, recoded on the interval 0–100.
(TIF)

S11 Fig Plot of betweenness centrality (BNC) values for *Hyalomma* adults. The size of the circles is proportional to BNC values, recoded on the interval 0–100.
(TIF)

S12 Fig Plot of betweenness centrality (BNC) values for *Ixodes* larvae. The size of the circles is proportional to BNC values, recoded on the interval 0–100.
(TIF)

S13 Fig Plot of betweenness centrality (BNC) values for *Ixodes* nymphs. The size of the circles is proportional to BNC values, recoded on the interval 0–100.
(TIF)

S14 Fig Plot of betweenness centrality (BNC) values for *Ixodes* adults. The size of the circles is proportional to BNC values, recoded on the interval 0–100.
(TIF)

S15 Fig Plot of betweenness centrality (BNC) values for *Rhipicephalus* larvae. The size of the circles is proportional to BNC values, recoded on the interval 0–100.
(TIF)

S16 Fig Plot of betweenness centrality (BNC) values for *Rhipicephalus* nymphs. The size of the circles is proportional to BNC values, recoded on the interval 0–100.
(TIF)

S17 Fig Plot of betweenness centrality (BNC) values for *Rhipicephalus* adults. The size of the circles is proportional to BNC values, recoded on the interval 0–100.
(TIF)

S1 Table The complete list of interactions (host preferences) of each tick genus and life stage (L, larvae; N, nymphs; A, adults) included in the analysis. Included also are the orders and families of the hosts.
(CSV)

S1 Data Betweenness centrality (BNC) values of each host family (converted to the range 0–100) for each tick genus and stage. (XLSX)
