# Supplementary figures and images for: Host preferences support the prominent role of Hyalomma ticks in the ecology of Crimean-Congo hemorrhagic fever
Source: PLoS Negl Trop Dis. 2018 Feb 8;12(2):e0006248. doi: 10.1371/journal.pntd.0006248 (PMC5821391; doi:10.1371/journal.pntd.0006248)

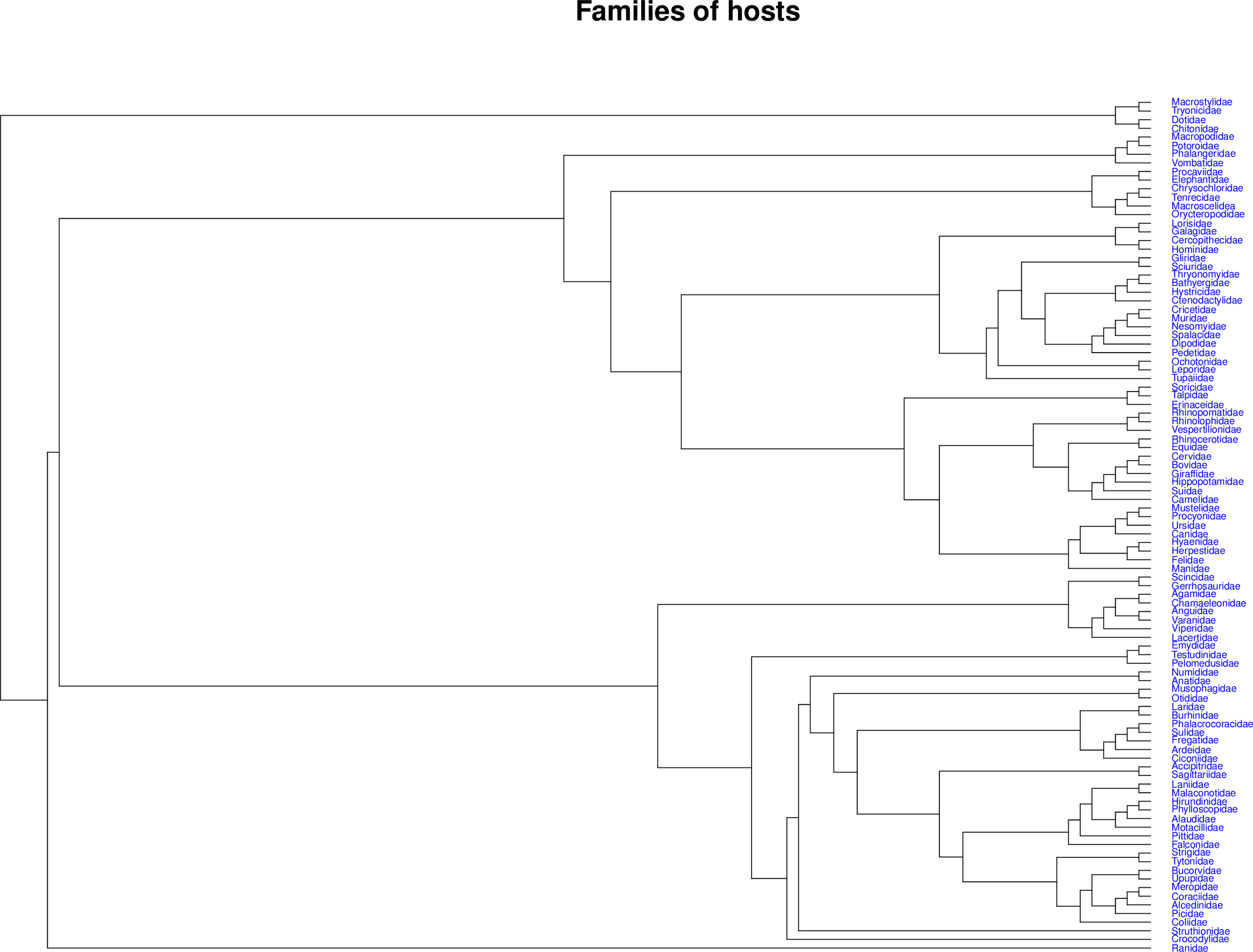

Supplement: S1 Fig — (TIF) [file pntd.0006248.s001.tif]

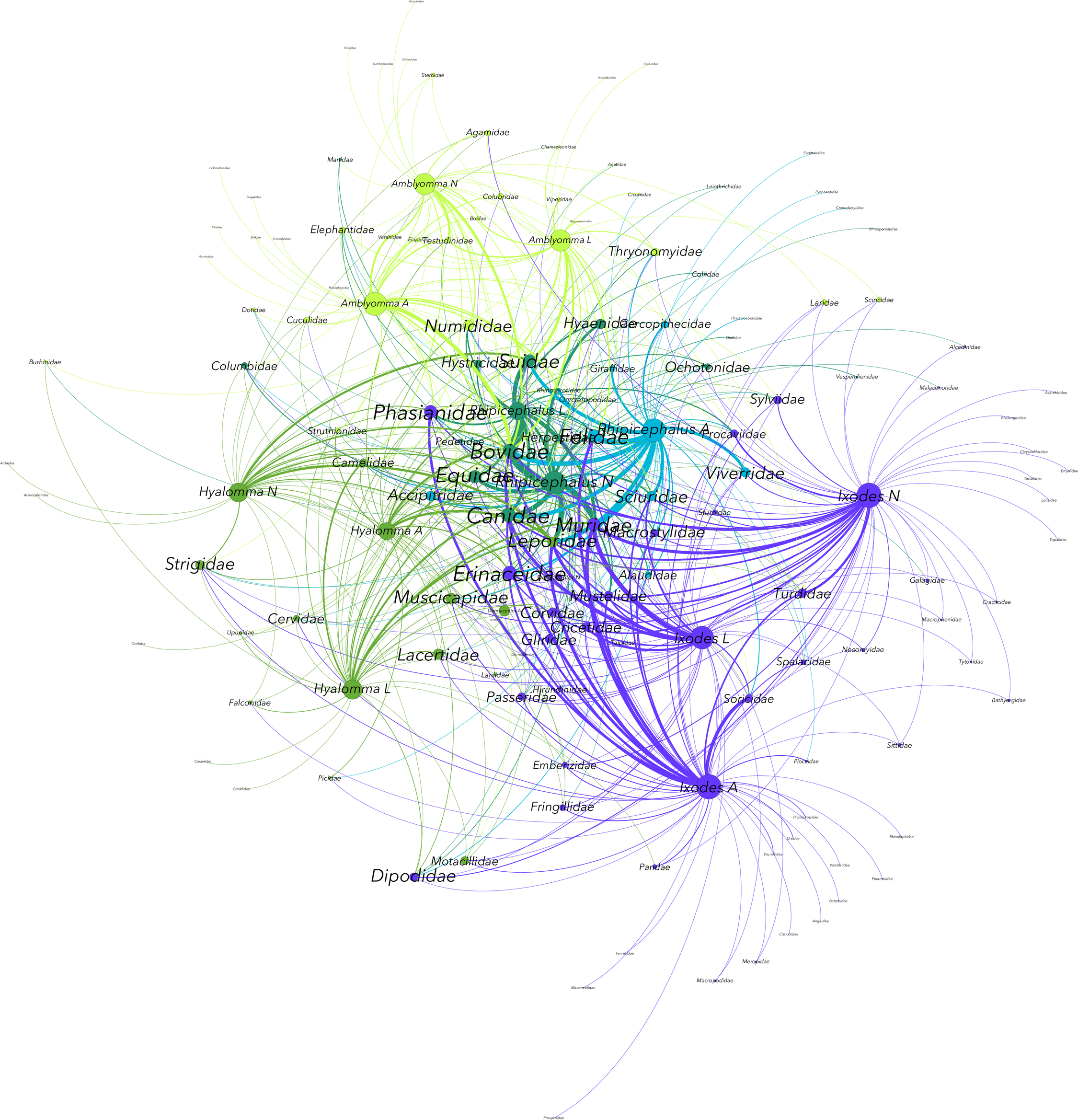

Supplement: S2 Fig — Nodes are sequentially colored to represent clusters, with nodes of the same color belonging to the same cluster. The size of each node is porportional to its betweenness centrality (BNC), and the size of the label is proportional to its weighthed degree (WD). The width of the links is propotional to the number of interactions recorded between hosts and ticks. The image can be resized and magnified on the screen for optimum readability of the names of host families with low WD. (TIF) [file pntd.0006248.s002.tif]

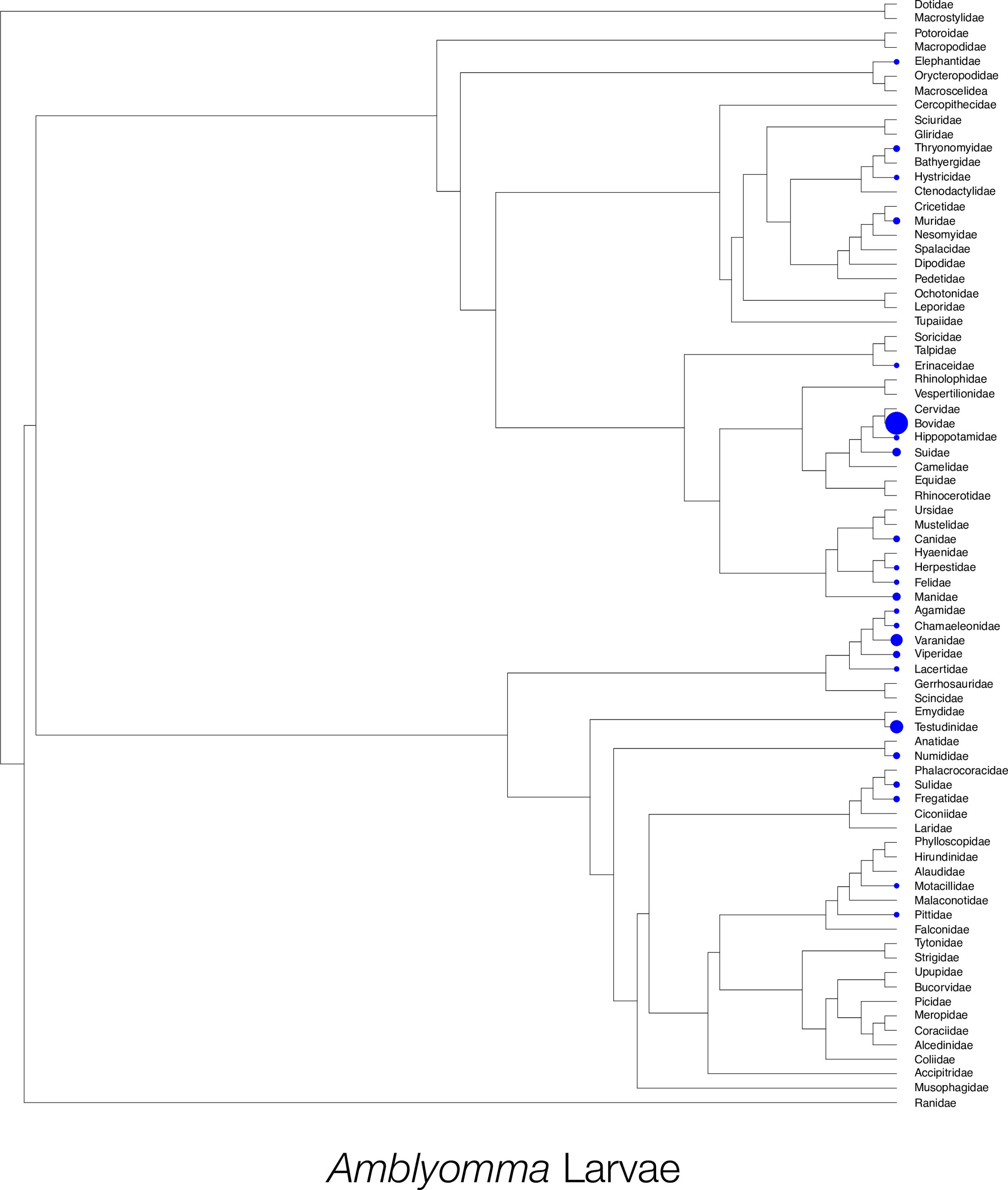

Supplement: S3 Fig — The size of the circles is proportional to BNC values, recoded on the interval 0–100. (TIF) [file pntd.0006248.s003.tif]

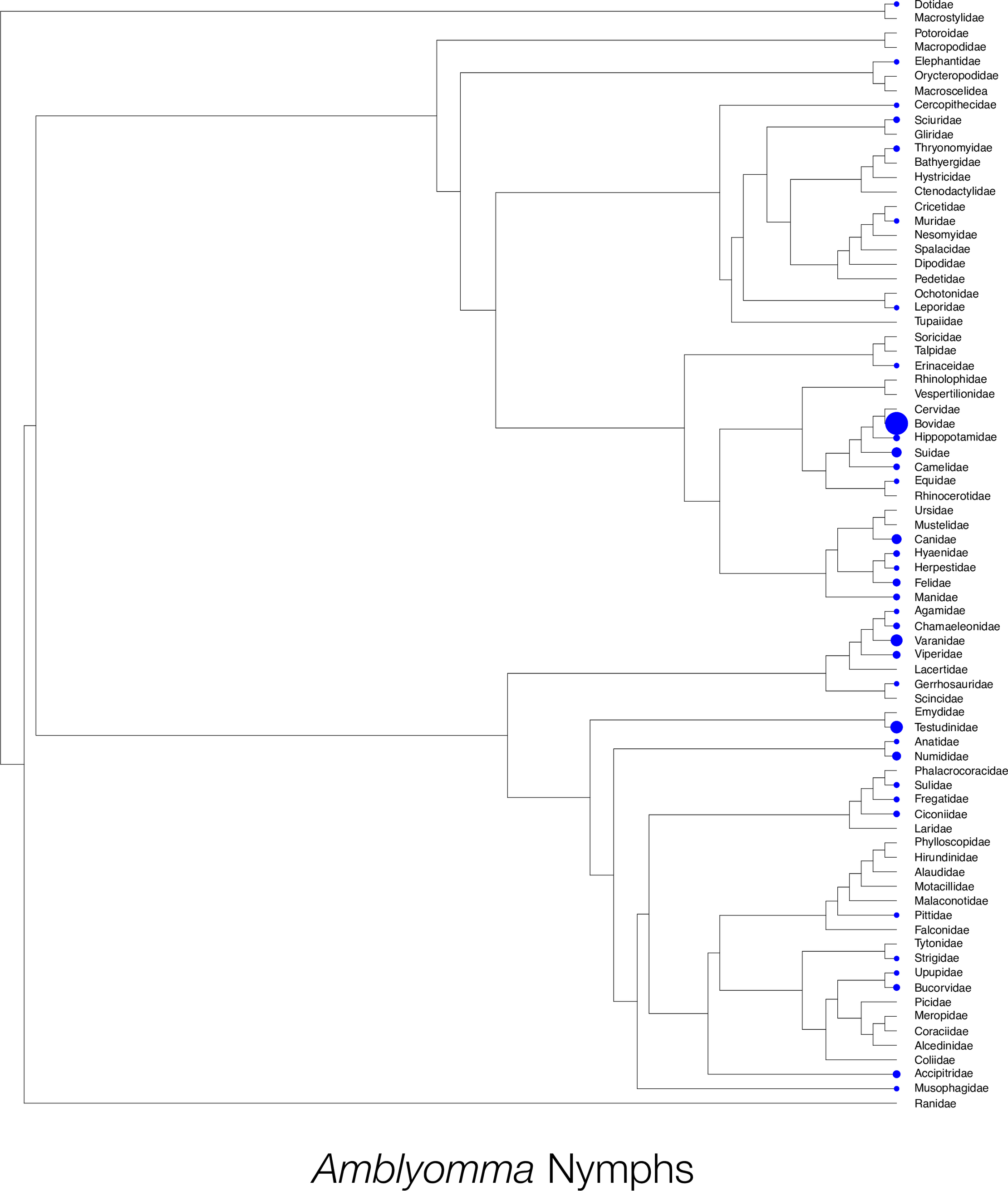

Supplement: S4 Fig — The size of the circles is proportional to BNC values, recoded on the interval 0–100. (TIF) [file pntd.0006248.s004.tif]

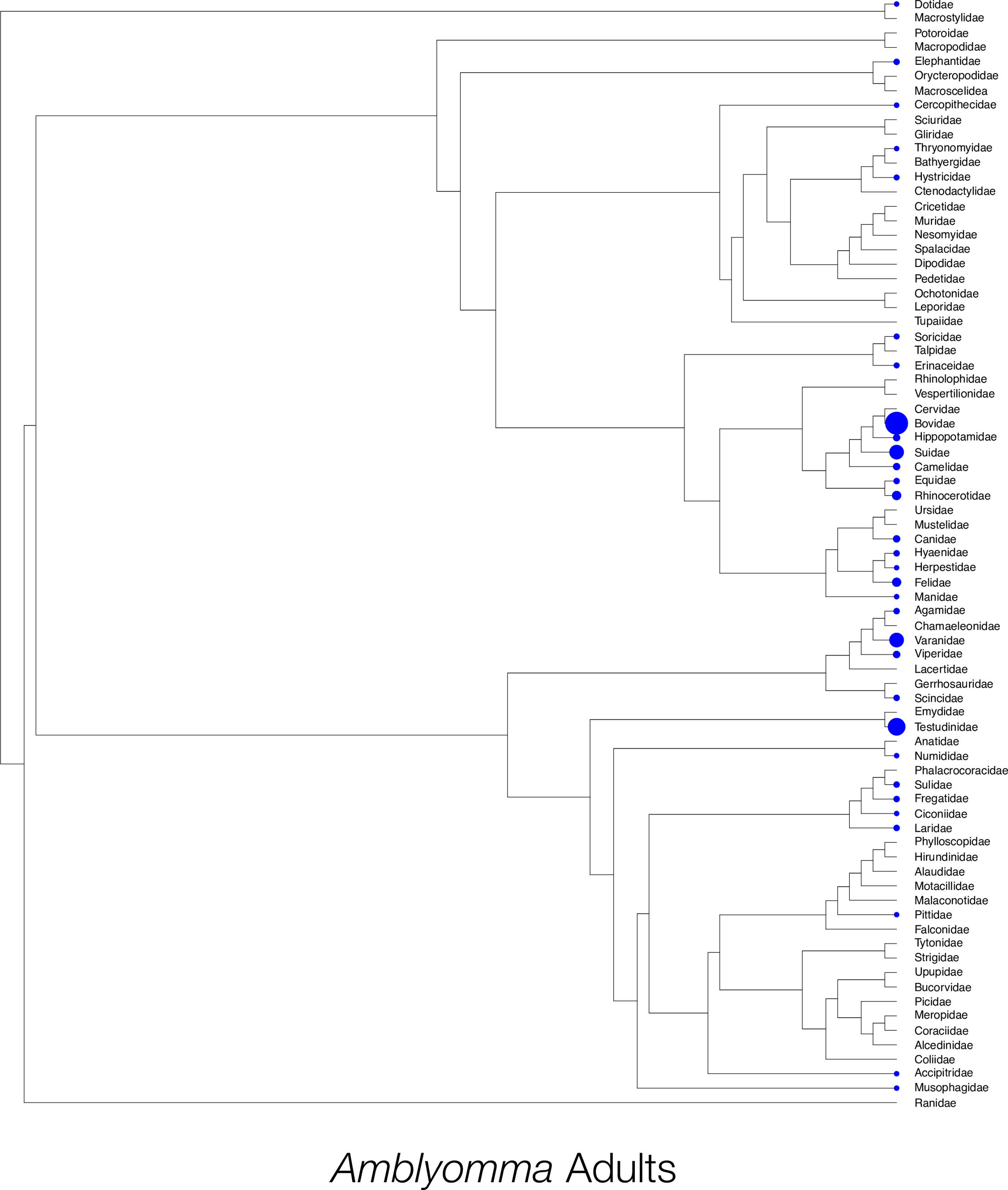

Supplement: S5 Fig — The size of the circles is proportional to BNC values, recoded on the interval 0–100. (TIF) [file pntd.0006248.s005.tif]

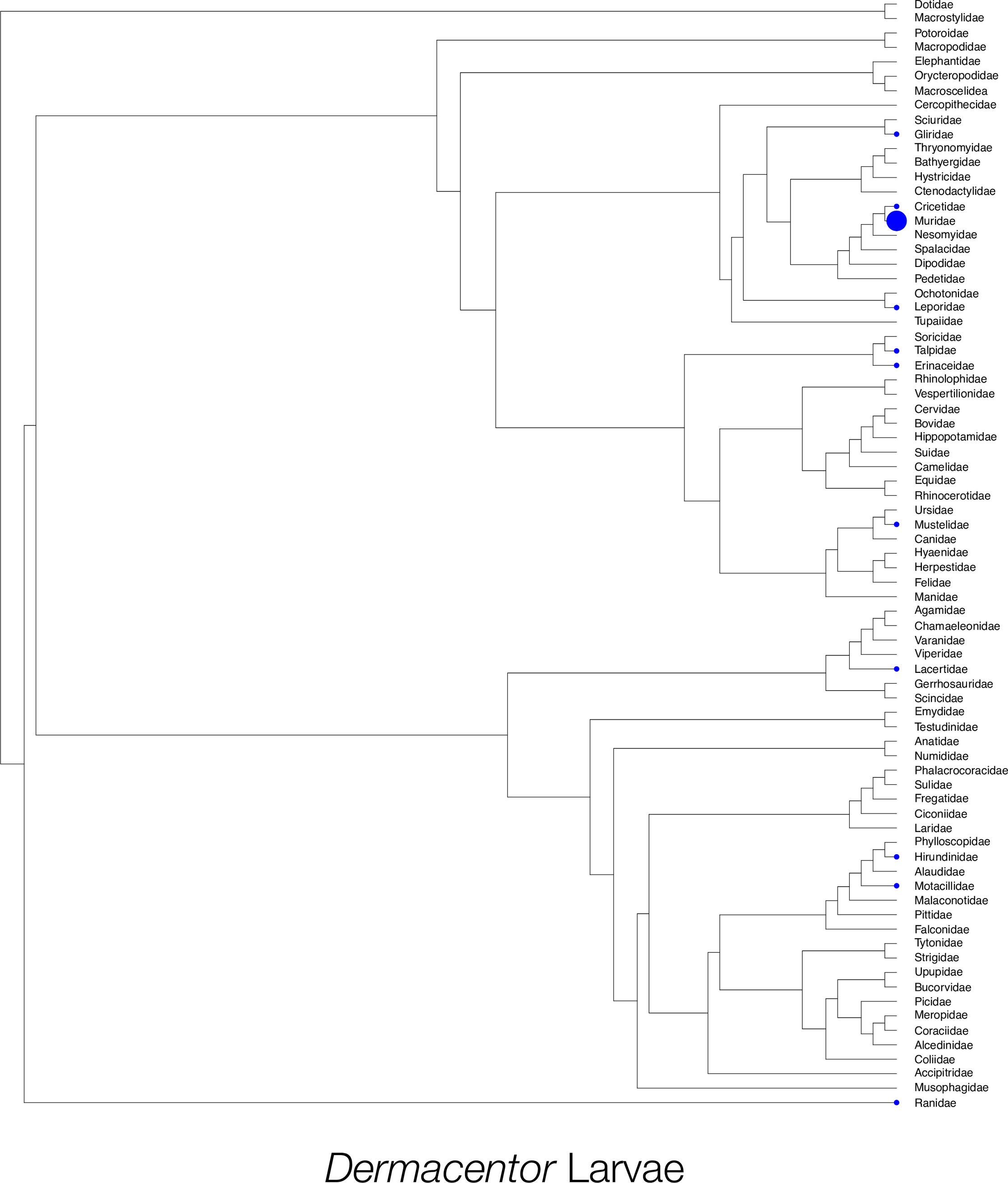

Supplement: S6 Fig — The size of the circles is proportional to BNC values, recoded on the interval 0–100. (TIF) [file pntd.0006248.s006.tif]

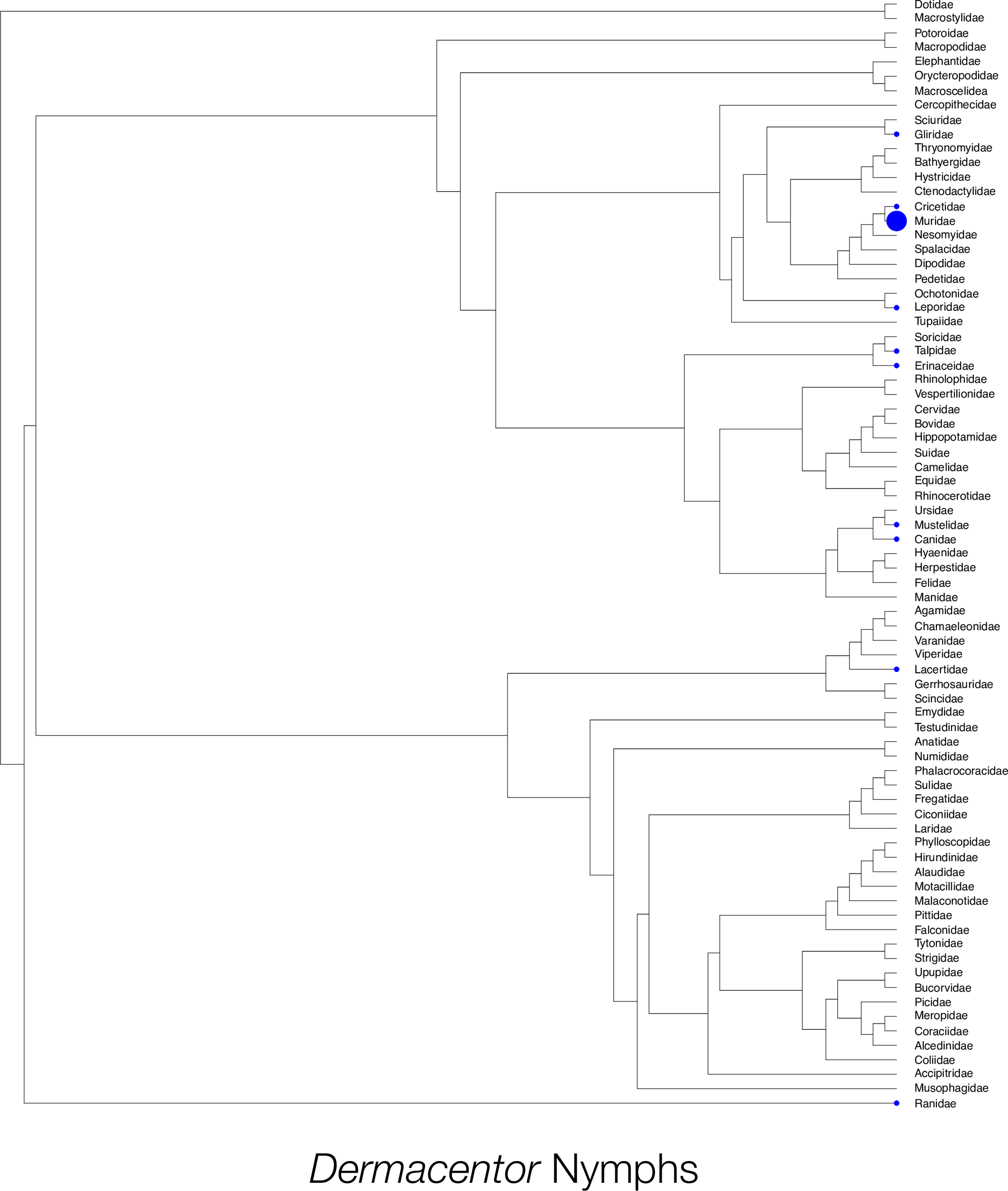

Supplement: S7 Fig — The size of the circles is proportional to BNC values, recoded on the interval 0–100. (TIF) [file pntd.0006248.s007.tif]

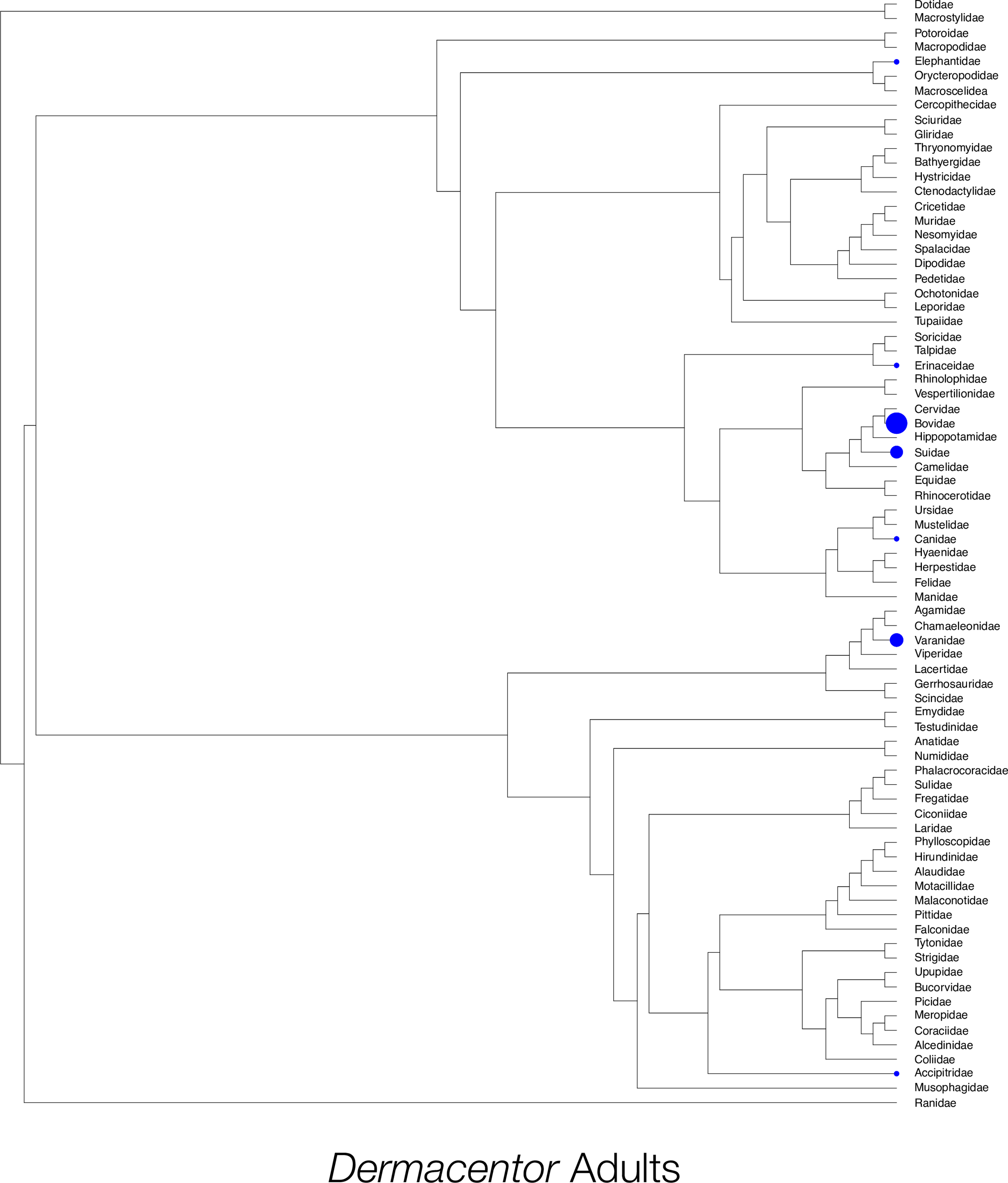

Supplement: S8 Fig — The size of the circles is proportional to BNC values, recoded on the interval 0–100. (TIF) [file pntd.0006248.s008.tif]

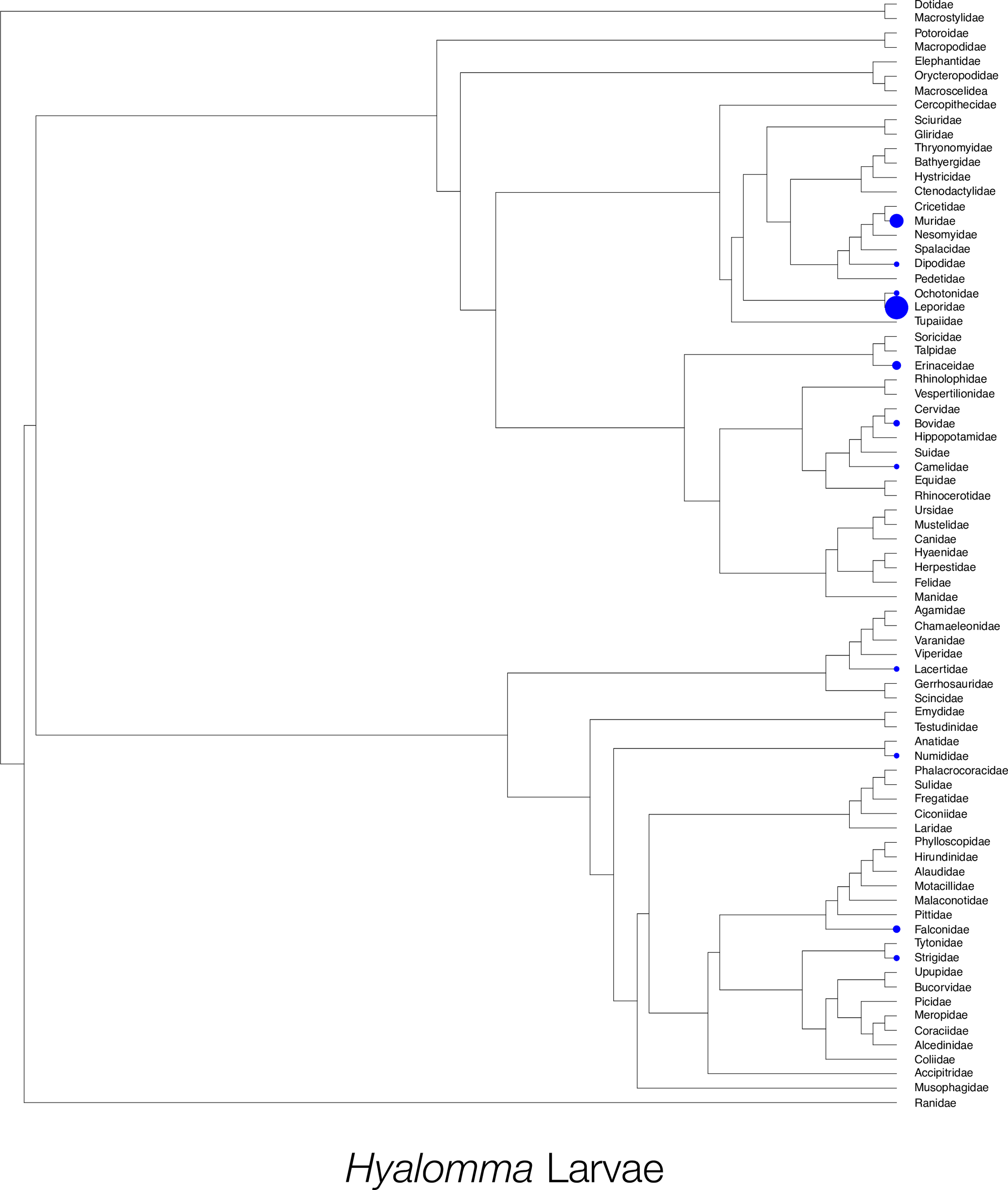

Supplement: S9 Fig — The size of the circles is proportional to BNC values, recoded on the interval 0–100. (TIF) [file pntd.0006248.s009.tif]

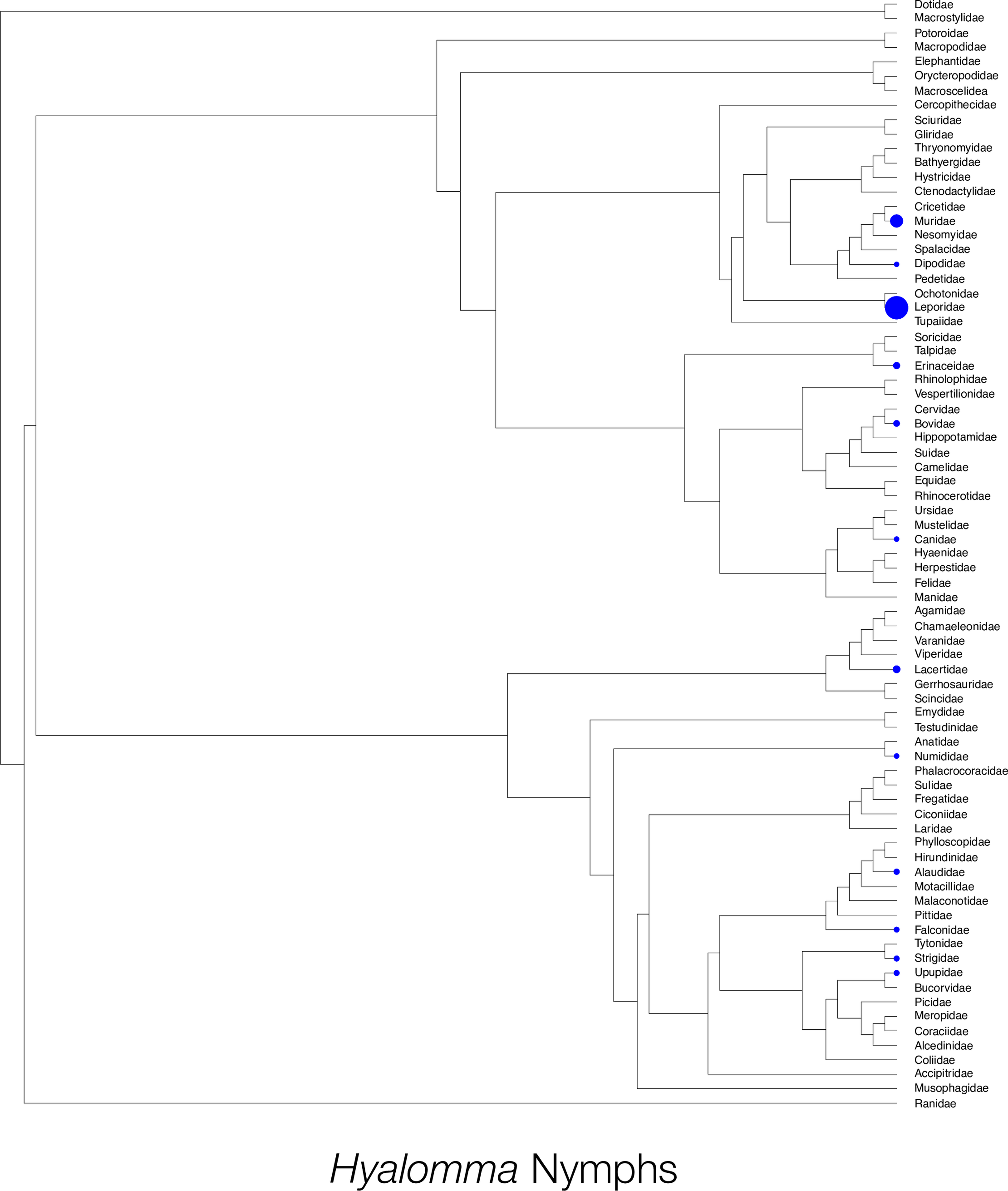

Supplement: S10 Fig — The size of the circles is proportional to BNC values, recoded on the interval 0–100. (TIF) [file pntd.0006248.s010.tif]

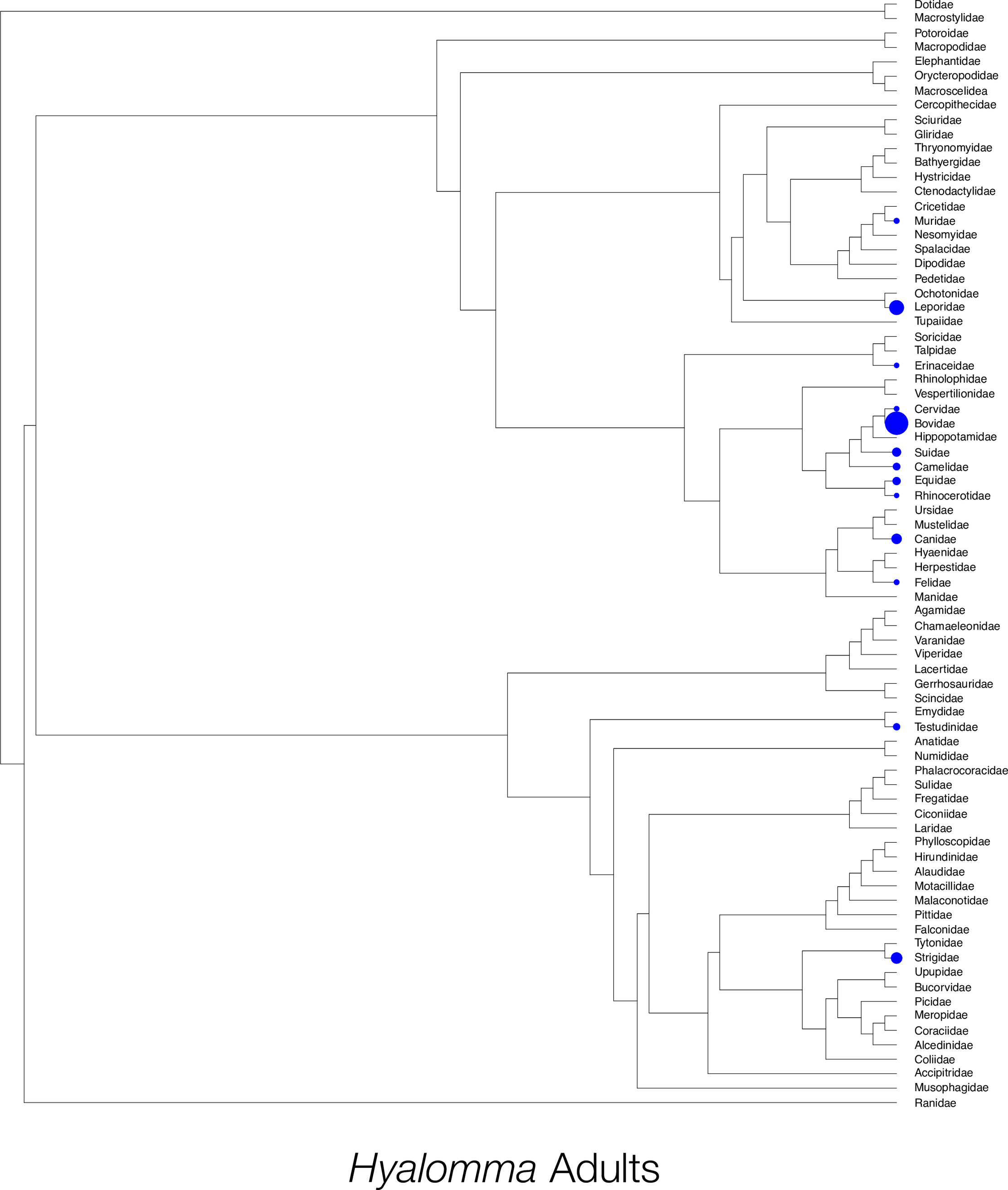

Supplement: S11 Fig — The size of the circles is proportional to BNC values, recoded on the interval 0–100. (TIF) [file pntd.0006248.s011.tif]

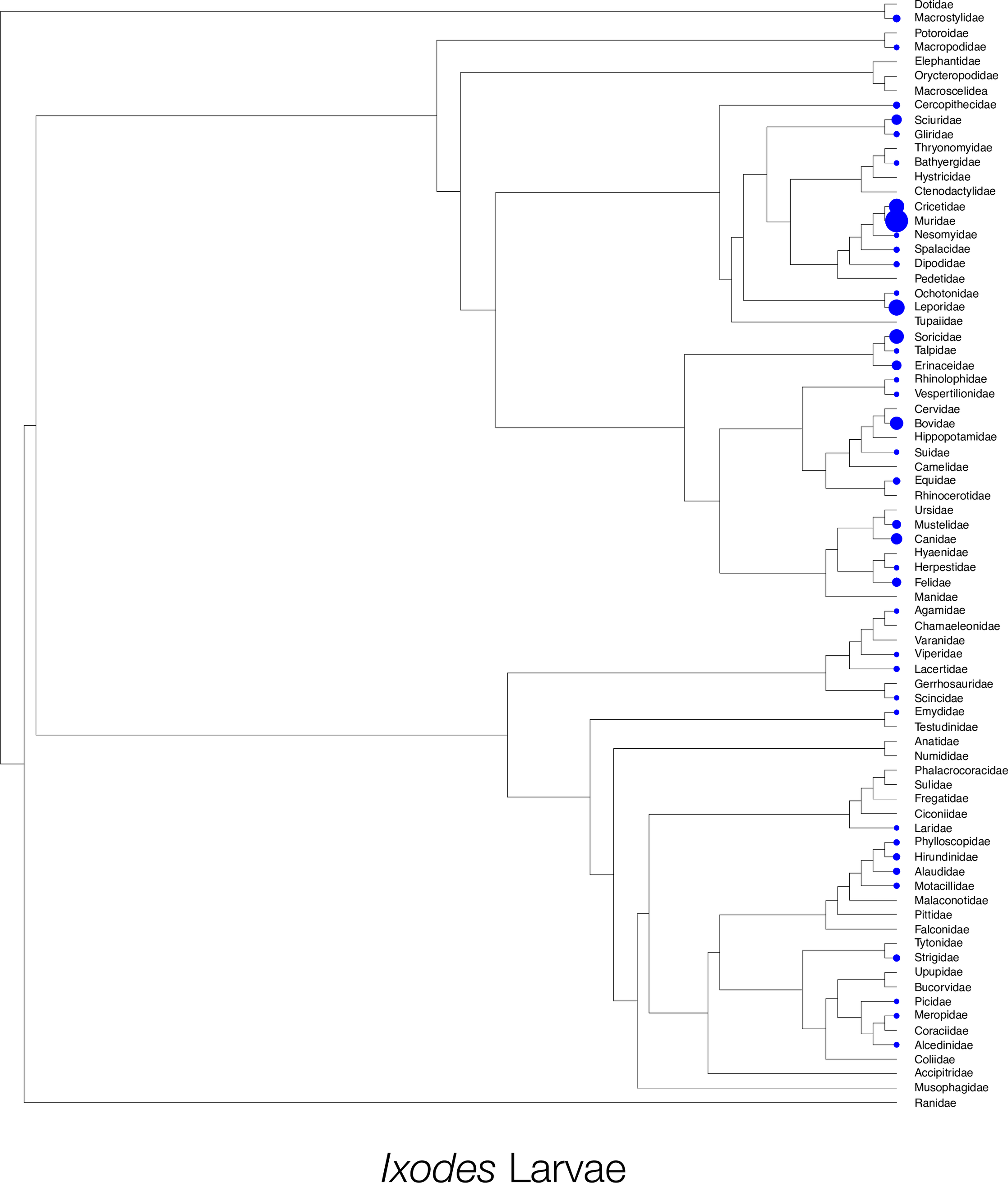

Supplement: S12 Fig — The size of the circles is proportional to BNC values, recoded on the interval 0–100. (TIF) [file pntd.0006248.s012.tif]

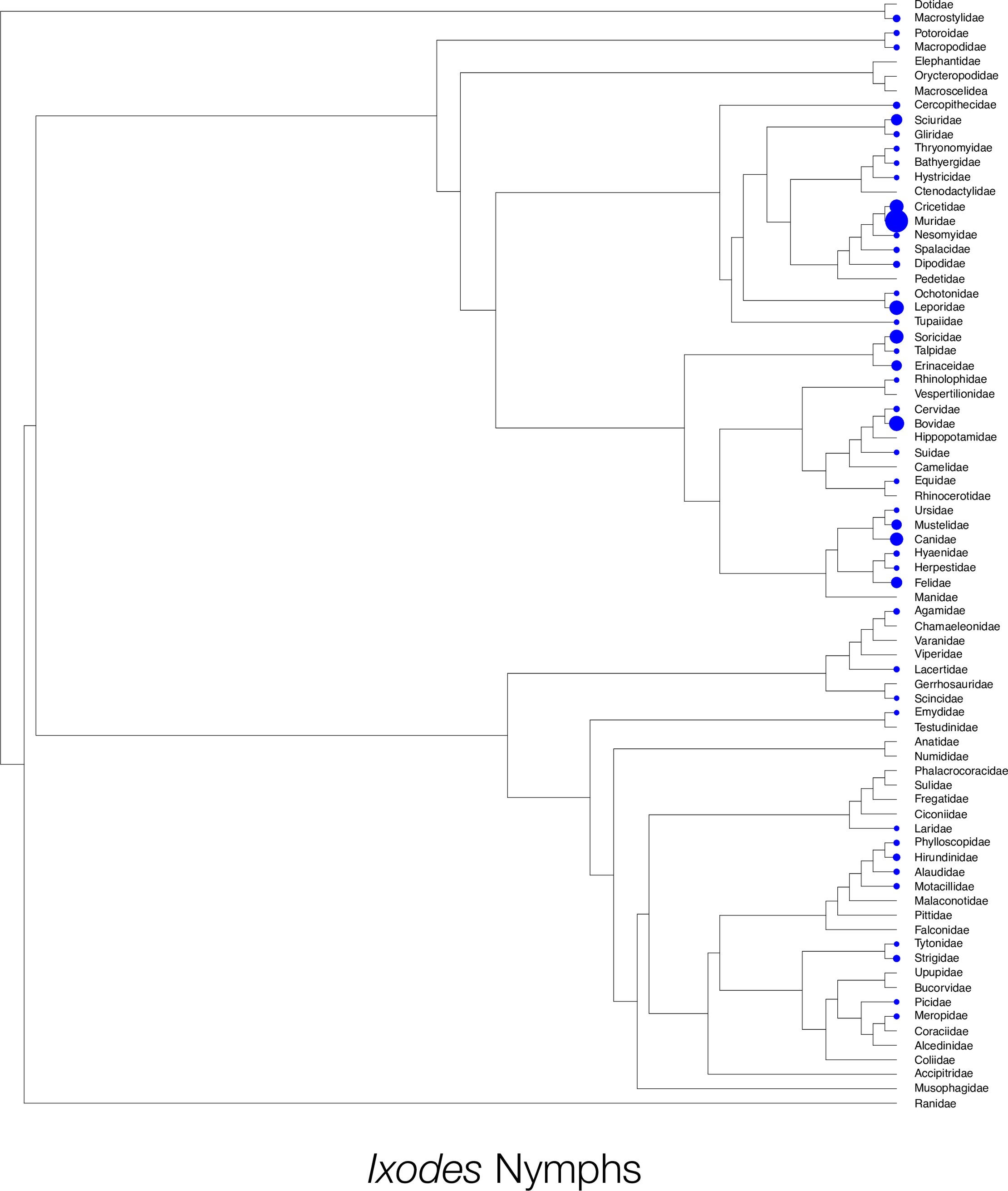

Supplement: S13 Fig — The size of the circles is proportional to BNC values, recoded on the interval 0–100. (TIF) [file pntd.0006248.s013.tif]

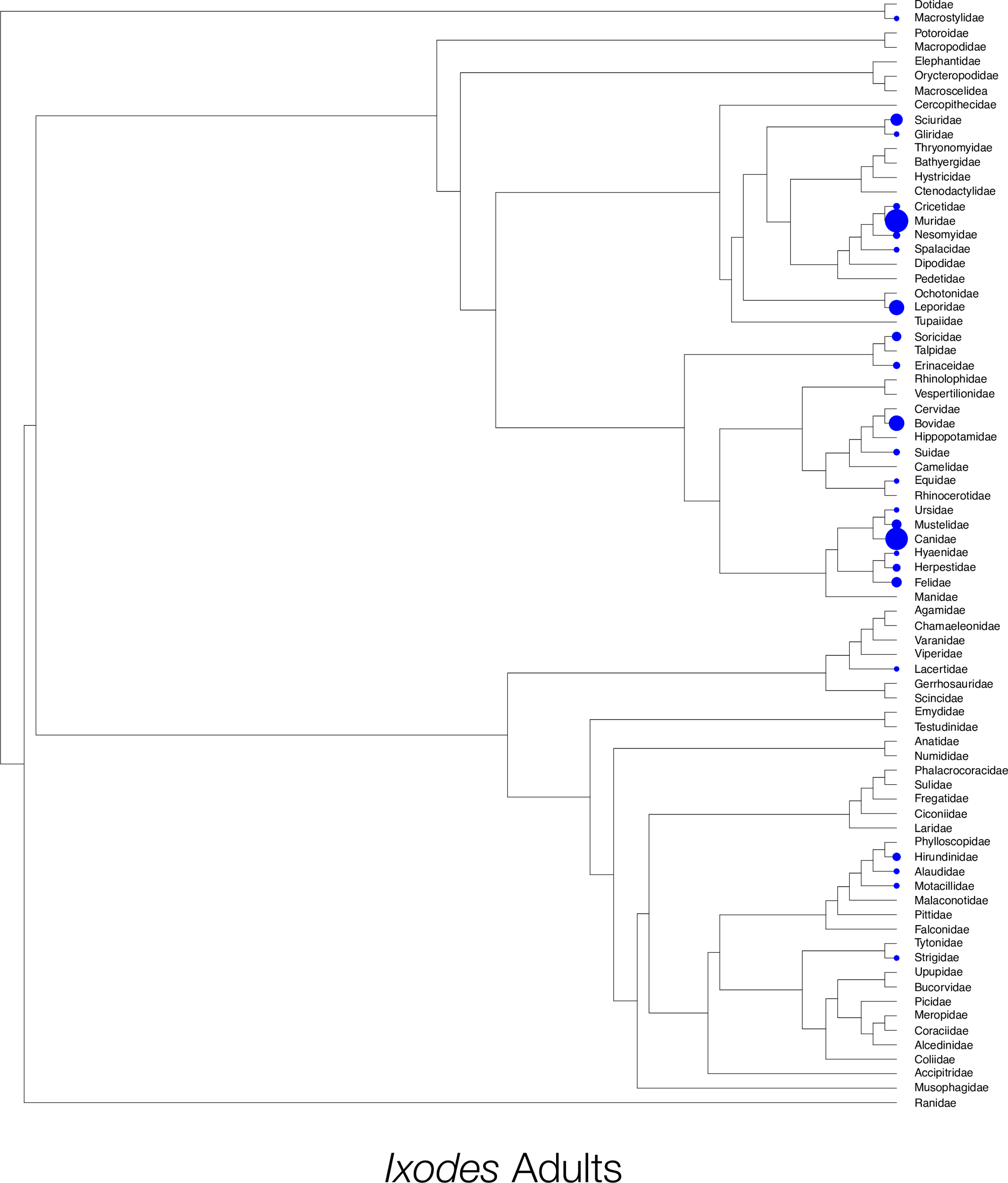

Supplement: S14 Fig — The size of the circles is proportional to BNC values, recoded on the interval 0–100. (TIF) [file pntd.0006248.s014.tif]

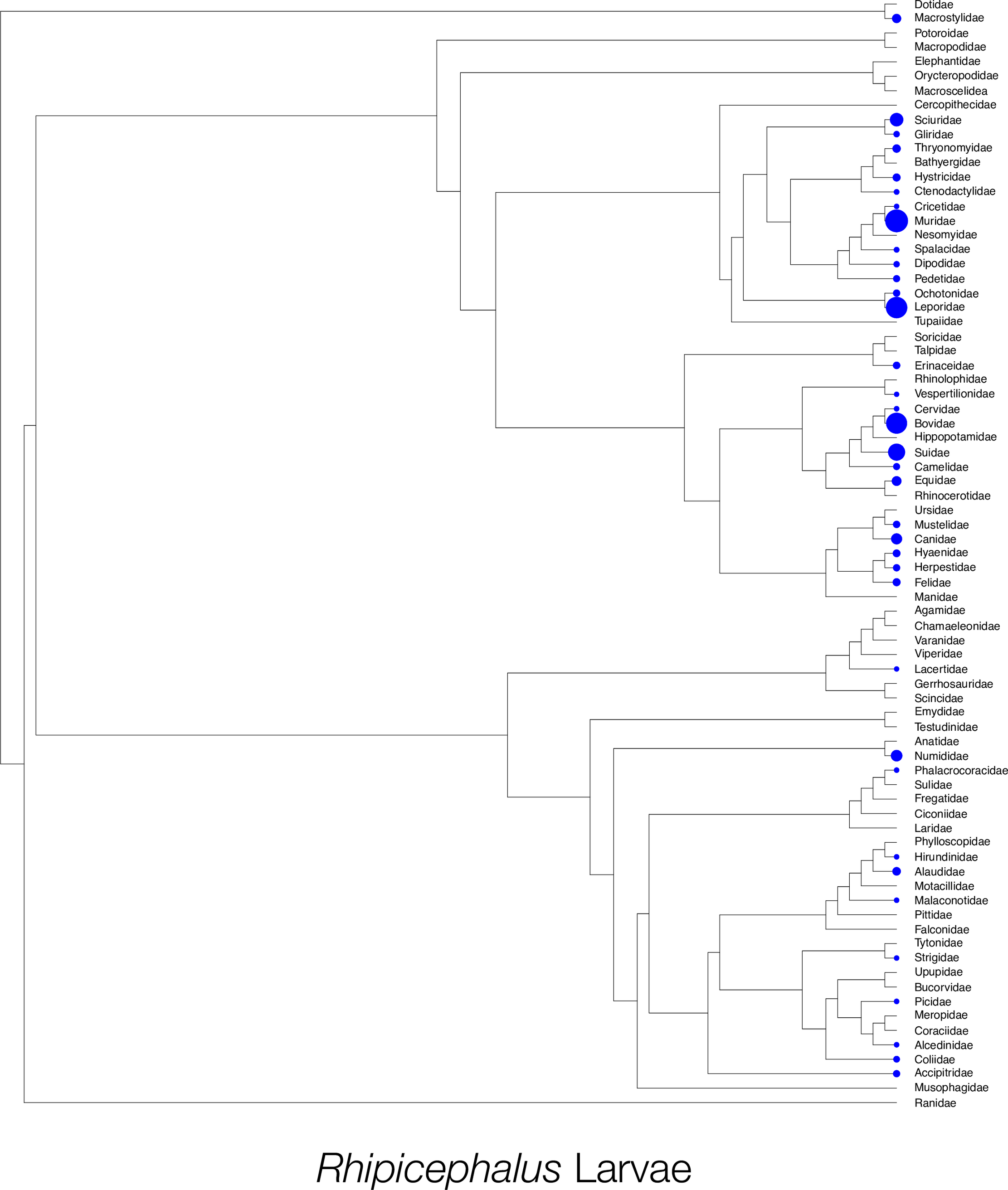

Supplement: S15 Fig — The size of the circles is proportional to BNC values, recoded on the interval 0–100. (TIF) [file pntd.0006248.s015.tif]

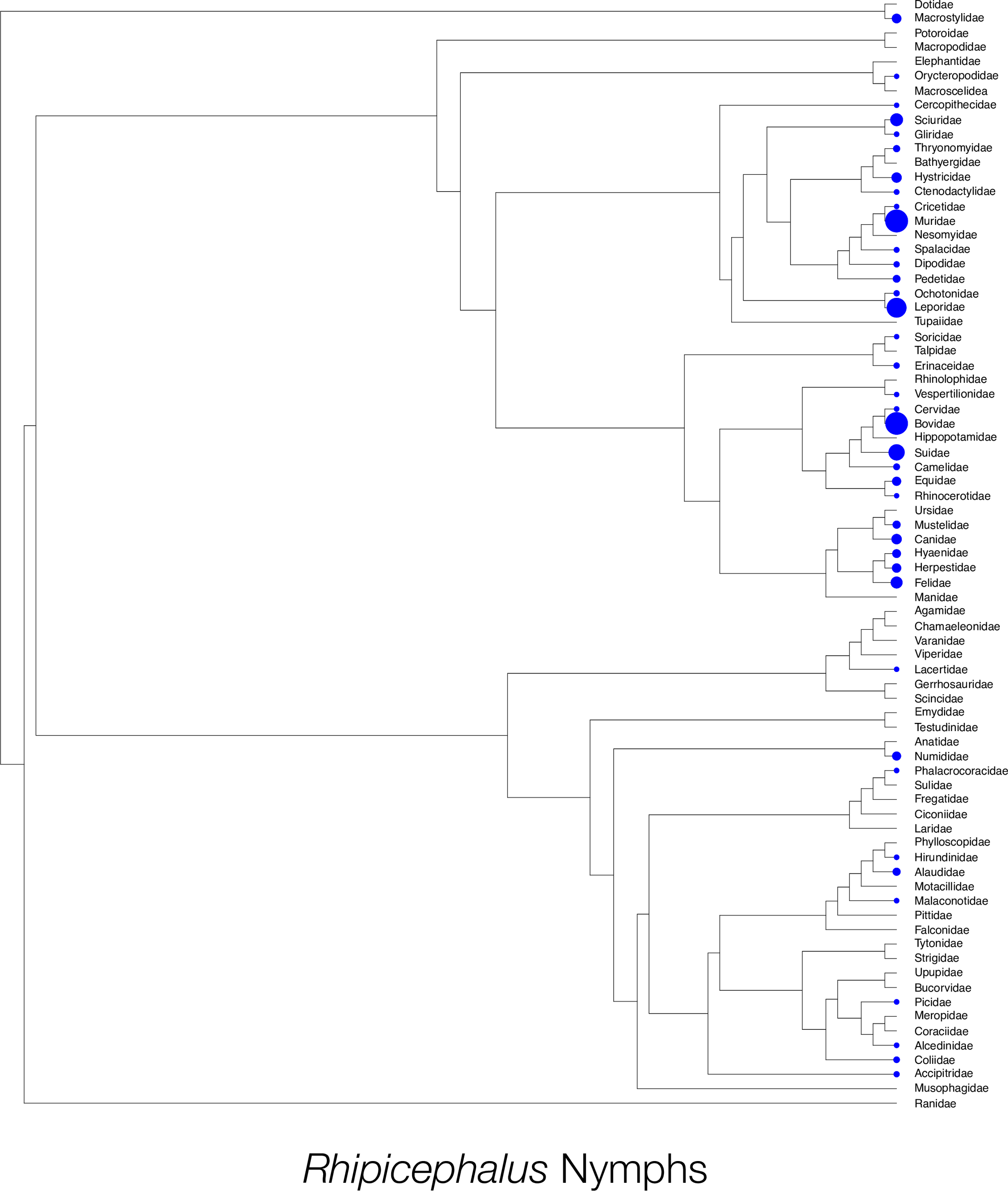

Supplement: S16 Fig — The size of the circles is proportional to BNC values, recoded on the interval 0–100. (TIF) [file pntd.0006248.s016.tif]

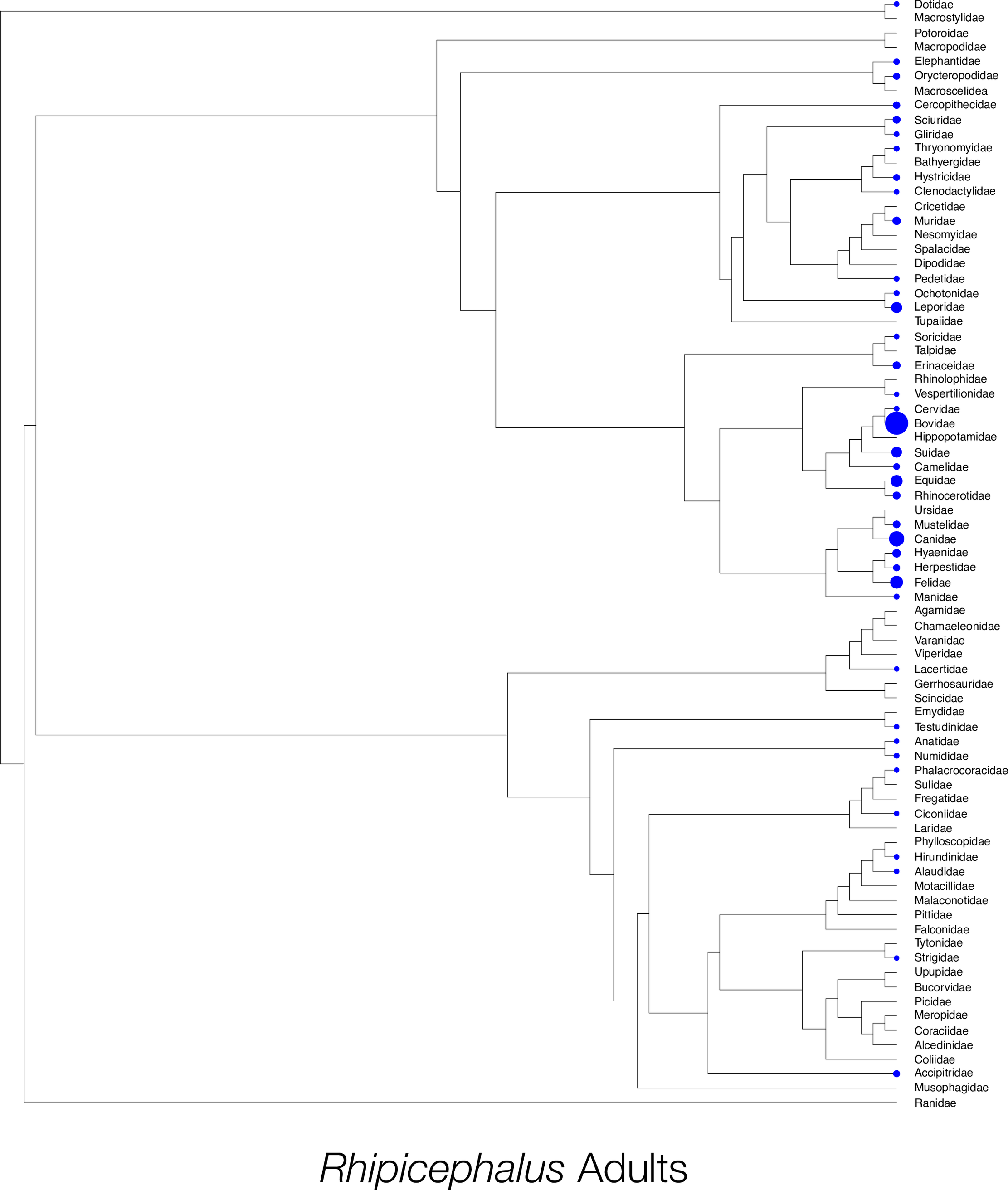

Supplement: S17 Fig — The size of the circles is proportional to BNC values, recoded on the interval 0–100. (TIF) [file pntd.0006248.s017.tif]
